# Shrink or expand? Just relax! Bidirectional grana structural dynamics as early light‐induced regulator of photosynthesis

**DOI:** 10.1111/nph.70175

**Published:** 2025-04-27

**Authors:** Joanna Wójtowicz, Radosław Mazur, Dainius Jakubauskas, Anna Sokolova, Christopher Garvey, Kell Mortensen, Poul Erik Jensen, Jacob J. K. Kirkensgaard, Łucja Kowalewska

**Affiliations:** ^1^ Department of Plant Anatomy and Cytology, Faculty of Biology University of Warsaw Miecznikowa 1 02‐096 Warsaw Poland; ^2^ Department of Metabolic Regulation, Faculty of Biology University of Warsaw Miecznikowa 1 02‐096 Warsaw Poland; ^3^ Department of Plant and Environmental Sciences University of Copenhagen Thorvaldsensvej 40 DK‐1871 Copenhagen Denmark; ^4^ Niels Bohr Institute, University of Copenhagen Universitetsparken 5 DK‐2100 Copenhagen Denmark; ^5^ Australian Nuclear Science and Technology Organisation Sydney NSW 2234 Australia; ^6^ Heinz Maier‐Leibnitz Zentrum (MLZ) Technische Universität München Lichtenbergstraße 1 85748 Garching Germany; ^7^ Department of Food Science University of Copenhagen Rolighedsvej 26 DK‐1958 Copenhagen Denmark

**Keywords:** chloroplasts, grana, light response, photosynthesis, thylakoids, ultrastructure

## Abstract

- Light‐induced structural changes in thylakoid membranes have been reported for decades, with conflicting data regarding their shrinkage or expansion during dark–light transitions. Understanding these dynamics is important for both fundamental photosynthesis research and agricultural applications. This research investigated the temporal sequence of thylakoid structural changes during light exposure and their functional significance.
- We combined high‐resolution structural approaches (transmission electron microscopy, confocal microscopy with 3D modeling, and small‐angle neutron scattering) with spectroscopic and electrophoretic analyses of the photosynthetic apparatus of *Arabidopsis thaliana* and *Ficus elastica* plants. A meta‐analysis of published ultrastructural data complemented our experimental approach to resolve existing contradictions.
- We discovered a three‐phase response pattern: initial shrinkage, expansion, and relaxation to dark‐state equilibrium. The initial shrinkage specifically regulated the cyclic/linear electron transport ratio, providing rapid photoprotection. We also showed that plants' acclimation to different light regimes modulates the kinetics of this response, with constant‐light‐grown plants exhibiting faster structural adaptations than those acclimated to glasshouse conditions.
- This work challenges the traditional binary model of light‐induced thylakoid structural dynamics, revealing a sophisticated temporal regulatory mechanism, with the dark‐adapted state serving as a relaxed equilibrium. The discovered three‐phase response reconciles decades of conflicting observations and reveals how plants achieve rapid photoprotection before engaging longer term adaptive responses.

## Introduction

Understanding how plants respond to light at different levels of their organization is essential to better comprehend the regulation of the photosynthesis process. Plant responses to light can be considered in the context of the initial chloroplast biogenesis, associated with the synthesis of the photosynthetic apparatus (Dubreuil *et al*., 2018; Armarego‐Marriott *et al*., 2019; Pipitone *et al*., 2021) and the short‐ and long‐term reactions of mature plants (reviewed by Johnson & Wientjes, 2020). The short‐term response of mature plants to light (timescale of minutes to hours) has been extensively studied at the macroscopic, organelle, nanomorphological, and molecular levels (summed up in Kirchhoff, 2019). While most studies across different scales on this topic lead to consistent conclusions, allowing us to understand the processes and mechanisms occurring during the transition from darkness to light, the existing literature on the nanomorphology of thylakoids presents a somewhat ambiguous picture. Illumination‐induced structural thylakoid dynamics on short timescales have been investigated by fluorescence microscopy (e.g. Herbstova *et al*., 2012; Wood *et al*., 2018), electron microscopy (e.g. Murakami & Packer, 1970; Yamamoto *et al*., 2013; Schumann *et al*., 2017; Wood *et al*., 2019; Li *et al*., 2020), atomic force microscopy (Clausen *et al*., 2014), light scattering (Murakami & Packer, 1971; Kirchhoff *et al*., 2011), and small‐angle scattering (Posselt *et al*., 2012; Nagy *et al*., 2013; Ünnep *et al*., 2014) methods, and different structural features of the thylakoid network were the prime interest in these studies.

In response to light, the ultrastructure of thylakoids undergoes transformations primarily aimed at optimizing the photosynthesis process, with particular emphasis on balancing cyclic electron transport (CET) and linear electron transport (LET; Höhner *et al*., 2020; Gu *et al*., 2022; Garty *et al*., 2024). The observed changes in the nanomorphology of thylakoids mainly concern the grana, which are the basic structural units of the internal thylakoid membrane network (Mazur *et al*., 2021). In the horizontal plane, as a result of illumination, the diameter of the grana decreases, shortening the transport path of electron carriers between Photosystem II (PSII) and Photosystem I (PSI) in the lateral space, thereby promoting LET (Wood *et al*., 2018). In the vertical plane of the grana, light can induce global transformations, which mainly involve a reduction in the number of membranes forming individual stacks (Rozak *et al*., 2002; Anderson *et al*., 2012). This leads to an increased number of double‐membrane appressed systems (the so‐called thylakoid doublets, earlier named membrane overlaps) compared with dark conditions (Gunning & Steer, 1975; Garty *et al*., 2024). Their role lies in enhancing LET efficiency by increasing the proportion of end thylakoids (Garty *et al*., 2024). In this case, the transport path of plastocyanin (PC) in LET is dramatically shortened, changing from a typical horizontal path to a vertical one, covering only the distance of the lumen (typically below 10 nm). This is related to the fact that end thylakoids are characterized by heterogeneous protein composition in which the stroma‐facing outer membrane of the end thylakoid exhibits a protein composition identical to stroma thylakoids rich in PSI, while the inner membrane facing the neighboring grana thylakoid exhibits a composition characteristic of PSII‐rich stacked regions (Wietrzynski *et al*., 2024).

Besides the reorganizations of grana in the vertical plane described above, light induction also leads to fine‐tuning the nanomorphology of individual thylakoids characterized by changes in the partition gap and lumen size (range of a few to several nanometers). In general, changes in the size of these two grana subcompartments result in changes in grana periodicity – the so‐called stacking repeat distance (SRD; Kirchhoff *et al*., 2011). Fluctuations in the size of grana subcompartments in response to light stimulus have been reported since the 1970s (see references for Supporting Information Tables S1, S2). The common denominator of the conducted studies was primarily the determination of two distinguished stages: dark vs light and the comparison between them. However, the diversity concerned the applied species, plant growth conditions before dark adaptation, type of material subjected to irradiation (leaves vs isolates), intensity and color of the light used, and the applied method of ultrastructure registration. In Tables S1 and S2, we summarized the diversity of applied experimental setups along with the demonstration of changes in SRD values for experimental works conducted over the last 50 yr.

For SRD dynamics, two seemingly irreconcilable processes are observed experimentally: illumination‐induced decrease in SRD and its expansion (Table S2). These SRD changes originated from both lumen and partition gap changes in both directions and with different magnitudes for the experimental setup and method used (Tables S1, S2). Furthermore, grana margin SRD expansion and a simultaneous shrinkage of grana core SRD have also been documented (Puthiyaveetil *et al*., 2014; Yoshioka‐Nishimura *et al*., 2014). Minor changes in thylakoid membrane thickness (*c*. 0.3 nm upon light exposure) represent an additional, albeit small, component affecting the overall SRD values (Johnson *et al*., 2011; Li *et al*., 2020).

The observed fine‐tuning of the grana structure in the vertical plane results from molecular processes primarily localized in distinct subcompartments (lumen and partition gap). While these processes can be analyzed separately to better characterize structural changes, their contributions to grana compression and overall changes in the SRD are inherently interconnected. Both subcompartments undergo distinct changes that collectively determine the overall structural dynamics of the grana.

The lumen, once seen as a simple ‘bag of ions’, is now recognized as a complex subcompartment containing both ions and soluble proteins at concentrations of *c*. 20 mg ml^−1^ (Kieselbach *et al*., 1998; Farci & Schröder, 2023). Its size and structure are particularly crucial for PC transport, especially considering the protrusion of membrane proteins (such as PSII complex extending 5 nm) into the lumenal space (Kirchhoff *et al*., 2011; Höhner *et al*., 2020; Graça *et al*., 2021). Even small changes in a few nanometers can affect PC diffusion efficiency or block it entirely, which can shift the proportion of LET to CET toward the latter (Höhner *et al*., 2020; Gu *et al*., 2022). Dynamic changes in lumen size occur primarily through osmotic water fluxes across thylakoid membranes, triggered by lumen acidification and ion movements (discussed in Gu *et al*., 2022; Hoh *et al*., 2024). These changes help regulate the proton motive force (pmf) and its components (electrical (Δψ) and chemical (ΔpH)), affecting nonphotochemical quenching (NPQ) and ATP synthesis (Li *et al*., 2004; Niyogi *et al*., 2004).

The partition gap (or stromal gap), measuring 4–5 nm between lipid bilayers, is crucial for maintaining grana structural integrity. Its size changes contribute directly to grana compression through the balance of three main forces: electrostatic repulsion, van der Waals attraction, and hydrostatic repulsion (Puthiyaveetil *et al*., 2017; Moazzami Gudarzi *et al*., 2021; Müh *et al*., 2021). The regulation of these forces depends heavily on membrane composition and ion distribution in the aqueous region of the partition gap. The thylakoid membrane exhibits a net negative charge, estimated between −25 and −63 mC (Barber, 1982; Puthiyaveetil *et al*., 2017). This is primarily due to the presence of proteins with negatively charged carboxyl groups, particularly light‐harvesting complex (LHC) proteins, which face the stromal side of the membrane. Additionally, posttranslational modifications of proteins can significantly alter the membrane's net charge. Among these modifications, light‐activated phosphorylation of the antenna and core photosynthetic proteins, which increases the membrane's negative charge, is considered crucial (Puthiyaveetil *et al*., 2017). There are two competing theories explaining protein interactions across this gap: One suggests electrostatic interaction between negatively charged stromal surfaces and positive charges on flexible N‐terminal segments of LHCs, and the other emphasizes the role of electric dipoles and van der Waals attraction (Kim *et al*., 2005; Barros *et al*., 2009; Albanese *et al*., 2020).

In contrast to the negatively charged membrane facing the partition gap, its aqueous region is rich in positive ions, with Mg^2+^ and K^+^ playing a dominant role (reviewed in Kaňa & Govindjee, 2016). It is not entirely clear whether positive ions merely screen the negative membrane charge through electrostatic interactions without direct charge neutralization, enabling effective van der Waals‐operated membrane attraction, or whether they directly bind to membrane proteins, forming strong salt bridges while ‘sandwiched’ between thylakoids (Wan *et al*., 2014).

This work investigates several fundamental questions regarding thylakoid membrane dynamics in response to light exposure. First, we examine whether the light‐induced structural modification of grana stacks in their vertical plane is a simple binary response (dark vs light states), as commonly portrayed in the literature, or whether it follows a more complex temporal pattern. Second, we investigate what biochemical and biophysical mechanisms might underlie these structural transitions, and how they differ between Arabidopsis (*Arabidopsis thaliana* (L.) Heynh.) and Ficus (*Ficus elastica* Roxb. ex Hornem) plant species with different grana architectures. Third, we explore how structural changes in the vertical plane of grana correlate with photosynthetic electron transport regulation, particularly the balance between CET and LET. We hypothesized that the commonly accepted model of light‐induced thylakoid expansion is incomplete and that the temporal dynamics of grana structural changes play a crucial regulatory role in photosynthetic efficiency. To test this hypothesis, we employed complementary structural analysis techniques together with functional measurements to characterize thylakoid membrane responses across multiple timescales of light exposure. Our approach aimed to reconcile contradictory findings in the literature by examining how growing conditions and experimental methodologies influence the observed structural transitions.

## Materials and Methods

### Plant material and growing conditions

Variegated variants of Ficus (*Ficus elastica* Roxb. ex Hornem), Epipremnum (*Epipremnum aureum* (Linden & André) G.S.Bunting), and Dieffenbachia (*Dieffenbachia seguine* (Jacq.) Schott), as well as the Arabidopsis (*Arabidopsis thaliana* (L.) Heynh.) Columbia (Col‐0) ecotype, were grown to full maturity in soil under glasshouse conditions. Ficus, Epipremnum, and Dieffenbachia plants were bought from the local supplier and acclimated for at least 1 month in the glasshouse before the illumination experiment, while Arabidopsis plants were cultivated from seeds. Additionally, Arabidopsis Col‐0 plants were grown in soil for 3 wk in a 9 h : 15 h, light : dark photoperiod at 21°C under control light conditions of 150 μmol photons m^−2^ s^−1^. For scattering experiments, Ficus plants were obtained from the flower shop, and no light acclimation was performed. Arabidopsis Col‐0 plants were grown at 21°C with a 12 h : 12 h, light : dark cycle under 110–140 μmol photons m^−2^ s^−1^ to full maturity. Plants were dark‐adapted for 48 or 16 h before collecting the dark samples (details provided in the Results section). For illumination experiments, plants were exposed to white light at a photosynthetically active radiation (PAR) intensity of 450–500 μmol photons m^−2^ s^−1^, and samples were collected at subsequent time points during illumination.

### Transmission electron microscopy

Samples for transmission electron microscopy (TEM) were prepared by cutting pieces of *c*. 3 mm^2^ area from the middle of the green and pale parts of the leaves. Plant material was fixed in 2.5% (w/v) glutaraldehyde in 50 mM cacodylate buffer (pH 7.4) for 2 h, washed in the buffer, and placed in a 2% (w/v) OsO_4_ at 4°C in 50 mM cacodylate buffer (pH 7.4) for *c*. 12 h. The specimens were dehydrated in a graded acetone series and embedded in a low viscosity epoxy embedding medium and cut on a Leica UCT ultramicrotome. Ultrathin sections were examined using a JEM 1400 electron microscope (Jeol, Japan). Structural grana features were measured manually using the ImageJ software (Abramoff *et al*., 2004), following the definitions of each parameter provided in Mazur *et al*. (2021). For the calculation of lumen fraction, we used the FFT module of ImageJ.

### Small‐angle neutron scattering experiments and modeling

Small‐angle neutron scattering (SANS) was performed at the ‘BILBY’ instrument in a time‐of‐flight mode (ANSTO, Sydney, Australia; Sokolova *et al*., 2019). Experimental parameters were as follows: measuring time of 60 min, *q* range of 0.0035–0.26 Å^−1^ (neutron wavelength of 2–20 Å), and resolution of 7%. Dark‐adapted D_2_O‐infiltrated leaf samples were measured in sample holders with quartz windows filled with D_2_O in this sequence: 3 μmol photons m^−2^ s^−1^ ambient light, illuminated with 500 μmol photons m^−2^ s^−1^ with KL1500 LED cold white light for 1 h, including an initial 20‐min period for transmission measurements before the SANS data collection. We use the white parts of the Ficus leafs as background for all thylakoid containing samples as shown in Fig. S1(g). The SANS modeling was performed following Jakubauskas *et al*. (2019) and Jakubauskas *et al*. (2021); for details, see Methods S1 and Table S3.

### Confocal laser scanning microscopy (CLSM)

A modified Tape‐Arabidopsis Sandwich method was used to prepare samples of a single layer of mesophyll cells for chloroplast *in vivo* 3D analysis by confocal laser scanning microscopy (CLSM; Mazur *et al*., 2019). Chloroplasts were visualized with the help of a Nikon A1 MP microscope as described in Kowalewska *et al*. (2016) with the *z*‐axis step set at 60 nm and z‐stack collection time 5–7 min. After recording the dark‐adapted chloroplast image, the specimen was illuminated without moving from the microscope sample holder for 50 min at 450 μmol photons m^−2^ s^−1^ using Schott KL 2500 LCD as a light source. After illumination, the image of the same chloroplast was collected. As a control, the dark‐adapted chloroplast was scanned again after 50 min in darkness.

The collected data sets were deconvolved using the AutoQuant X3 software (Media Cybernetics Inc., Rockville, MD, USA), and the 3D models of Chl fluorescence surface were created based on the pixel gradient intensity algorithm using the Imaris v.8.4.2 software (Bitplane AG, Zurich, Switzerland).

### Thylakoid isolation

Thylakoid membranes were isolated from fresh leaves by homogenization in a buffered isotonic medium and subsequent centrifugations as described previously (Krysiak *et al*., 2024). The isolation procedure took *c*. 1 h. Buffers were supplemented with 10 mM NaF to prevent phosphatase activity. The concentration of Chl was quantified spectrophotometrically after extraction with 80% (v/v) acetone (Lichtenthaler, 1987).

### Sodium dodecyl sulfate‐polyacrylamide gel electrophoresis (SDS‐PAGE) and immunoblot

Proteins were separated by standard SDS‐PAGE electrophoresis and transferred on polyvinylidene fluoride membranes. Selected proteins were detected by primary antibodies: Lhcb1 (AS01 004), Lhcb1‐P (AS13 2704), Lhcb2 (AS01 003), Lhcb2‐P (AS13 2705), Lhcb4 (AS04 045), Lhcb5 (AS01 009), Lhca1 (AS01 005), D1 (AS10 704), D1‐P (AS13 2669), Atpβ (AS05 085), PsaB (AS10 695), acetylated lysine (AS15 3018) (all from Agrisera, Vännäs, Sweden), followed by the anti‐rabbit horseradish peroxidase conjugate (AS09 602; Agrisera), and the ECL Detection System (Bio‐Rad). Quantitative analysis was performed using the ImageJ software.

### Blue Native PAGE

Samples containing 15 μg of Chl were centrifuged at 7000 ***g*** for 5 min at 4°C. The pellet was gently resuspended in 24 μl of Native PAGE sample buffer (Invitrogen) followed by the addition of *n*‐Dodecyl‐β‐d‐maltoside and digitonin to a final concentration of 1% (w/v) of each. Samples were incubated with gentle shaking on a vortex for 30 min at 4°C in darkness, following the centrifugation at 18 000 ***g*** for 15 min at 4°C. The supernatant was mixed with Coomassie Brilliant Blue G‐250 to a final concentration of 1% (w/v). Samples containing 8.3 μg of Chl were loaded into gel wells, and the electrophoresis was run using 4–16% (w/v) gradient acrylamide gels (NativePAGE™; Invitrogen) according to the manufacturer's protocol (Invitrogen).

### Membrane negative charge measurements

To determine negative charge density on thylakoid membranes, a fluorescence method based on 9‐aminoacridine fluorescence was applied (Chow & Barber, 1980a,b). In low salt concentrations, 9‐aminoacridine fluorescence is quenched due to its interaction with the negatively charged membranes. Adding Mg^2+^ releases 9‐aminoacridine from the membrane, resulting in fluorescence rise; this provides information on the overall negative charge of the thylakoid membrane. Thylakoid samples were washed in 10 mM Hepes‐NaOH (pH 7.5) buffer containing 0.1 M sorbitol and 1 mM EDTA, and the pellet was resuspended in the washing buffer to a final Chl concentration of 1.5 mg ml^−1^. The 9‐aminoacridine fluorescence emission was monitored at 455 nm using a Shimadzu RF5301‐PC fluorometer. The obtained fluorescence traces were normalized to the maximal fluorescence signal (*F*_max_) in the presence of thylakoids. For details, see Methods S1. Due to method limitations described elsewhere (Bérczi & Møller, 1993), we did not calculate the negative charge density of the membrane; instead, we compared the *F*/*F*_max_ values for washed thylakoids.

### 77 K fluorescence emission measurements

Chl emission spectra were measured at 77 K from isolated thylakoids diluted to 10 μg ml^−1^ Chl in 20 mM Hepes‐NaOH buffer (pH 7.5) containing 330 mM sorbitol, 15 mM NaCl, and 4 mM MgCl_2_ using a modified Shimadzu RF‐5301PC spectrofluorometer where excitation and emission beams were guided by optical fibers. The spectra were collected in the range of 600–800 nm (1 nm interval) after excitation with a 440‐nm light beam. Recorded spectra were normalized to the same area under the spectrum.

### Simultaneous Chl *a* fluorescence and P700 measurements

Measurements were carried out using a Dual‐PAM 100 fluorometer (Heinz Walz GmbH) as described previously (Mazur *et al*., 2016). All measurements were performed on 16‐h dark‐adapted leaves. After the determination of *F*_0_, *F*_M_, and *P*_M_, the actinic light (450 μmol photons m^−2^ s^−1^) was on, and *F*_M_′ and *P*_M_′ values were measured by the application of a series of saturation pulses during 2 h with a 5‐min interval between two consecutive saturation pulses. The effective PSII quantum yield (Y(II)) was calculated according to Genty *et al*. (1989), and the photochemical quantum yield of PSI (Y(I)) was calculated according to Klughammer & Schreiber (2008b). For the estimation of the yield of cyclic electron transport (Y(CET)), the difference between Y(I) and Y(II) was calculated: Y(CET) = Y(I) – Y(II) according to Huang *et al*. (2010).

### P515 signal *in vivo* measurements

Measurements were carried out using a Dual‐PAM 100 fluorometer (Heinz Walz GmbH) equipped with the P515/535 emitter‐detector module. Dark–light–dark induced slow kinetics of the P515 signal were measured on 16 h dark‐adapted plants. Actinic light of 450 μmol photons m^−2^ s^−1^ was applied for 50 min, followed by 10 min of darkness. In the second experimental setup, the increasing intensity of actinic light (six steps: 15, 100, 300, 750, 1750, and 4000 μmol photons m^−2^ s^−1^) was applied; each 3‐min illumination step was followed by 5 min of darkness. The pmf and the relative amplitudes of Δψ and ΔpH were calculated according to Klughammer & Schreiber (2008a) and references therein.

### Statistical analysis

The statistical significance was verified by one‐way ANOVA with the *post hoc* Tukey test at *P* = 0.05. The number of repetitions of specific experiments is given in the figure legends.

## Results

### Grana stack size and structural dynamics: a multi‐method approach

To address the topic of the structural dynamics of thylakoid grana in a vertical direction, we decided to select plants that naturally exhibit significantly different quantitative organization of stacked membranes (Mazur *et al*., 2021). This approach allowed us to investigate whether structural changes focused on fluctuations in the membrane periodicity could depend on grana size. As a species with relatively small grana stacks, we chose the model plant Arabidopsis. We paired it with a species possessing the so‐called ‘giant grana stacks’ characterized by exceptionally large diameter and height. To select a species with large grana, we tested three shade‐tolerant plants (Dieffenbachia, Ficus, Epipremnum) that exhibit ‘variegated’ patterns – meaning part of their leaf blade lacks chloroplasts (pale areas; Fig. S1a–d). Choosing a species from the ‘variegated’ group allowed us to rigorously analyze results obtained via SANS, where the pale areas served as a reference sample for extracting signals characteristic of thylakoids. Our TEM analyses showed that Ficus had the largest area of grana stacks among the studied plants (Fig. S1e,f), and following SANS analysis indicated the possibility of registering signals corresponding to thylakoids in this species (Fig. S1g). Consequently, we chose both Arabidopsis and Ficus for further studies aimed at understanding the structural dynamics of grana under light influence.

In the initial stage of our research, we applied a simple experimental model in which we conducted observations at two time points corresponding to two distinct plant states following the most commonly used thylakoid illumination setups (Tables S1, S2). We analyzed plants adapted to darkness and those after exposure to intense light for 50 min (450 μmol photons m^−2^ s^−1^). In our first experimental setup, we used plants dark‐adapted for 48 h to establish a clear distinction between dark and light states of thylakoid membranes. We performed a multifaceted analysis of the thylakoid structure in this setup using three complementary methods: (1) CLSM allowed us to observe structural changes in thylakoid networks over time in selected chloroplasts, albeit with relatively low resolution *in vivo*; (2) TEM enabled high‐resolution analyses of fixed samples collected at two analyzed time points; and (3) SANS provided high‐resolution and large length scale analyses of living tissue, although without direct visualization.

**Fig. 1:**
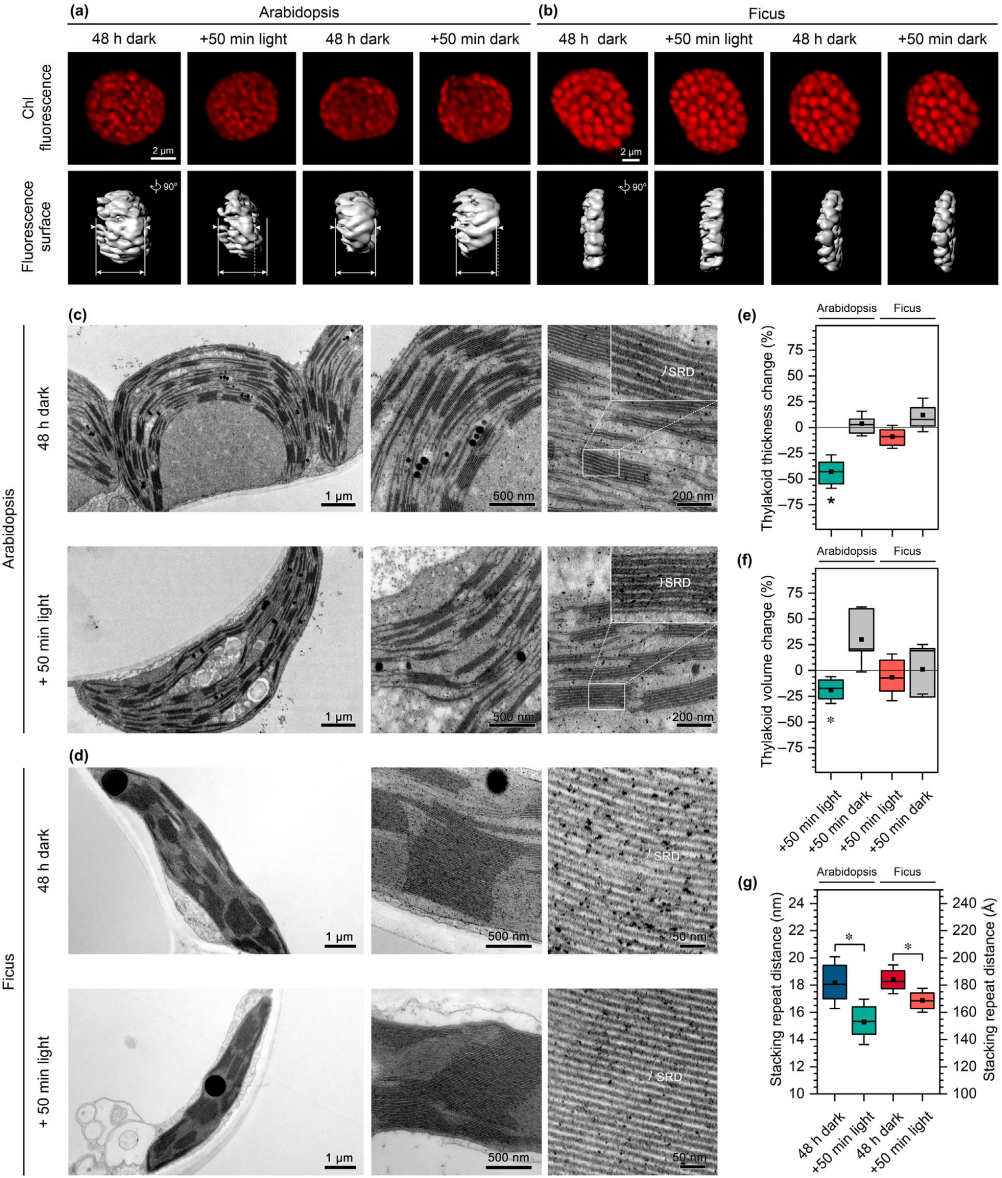
Light‐induced changes in the stacking repeat distance of typical size and giant grana stacks visualized by confocal laser scanning microscopy (CLSM) and transmission electron microscopy (TEM). (a, b) Images of Chl fluorescence of chloroplasts visible inside of living mesophyll cell of Arabidopsis (a) and Ficus (b) with 3D isosurface generated and rotated to visualize thickness of the network in the vertical plane of chloroplast; pairs of representative images present a particular chloroplast before and after 50 min of illumination or an additional 50 min of darkness (control); all images in (a) and (b) are in the same scale. (c, d) Electron micrographs of mesophyll chloroplasts of Arabidopsis (c) and Ficus (d) before and after 50 min of illumination; representative chloroplast images (left side), thylakoid network enlargements (middle part), and grana structural details (right side). (e–g) Quantitative changes in selected thylakoid structural parameters obtained from CLSM (e, f) and TEM (g) data. CLSM data were obtained from at least six biological replicates, and TEM data were obtained from three biological replicates. The bottom and top of each box represent the 25 and 75 percentiles, respectively. The error bars denote SD, horizontal line, and square mark median and mean value, respectively. Pairs of results marked with an asterisk differ significantly at *P* ≤ 0.05 (one‐way ANOVA with the *post hoc* Tukey test; *n* = 25–66 (e), 6–16 (f), 93–110 (g)).

The results obtained from TEM and CLSM methods were consistent and indicated thylakoid compression in the vertical plane due to 50 min of light exposure (Fig. 1). These changes were particularly pronounced in Arabidopsis, which exhibited lower granum heights than Ficus. Statistical analysis of CLSM data revealed significant thylakoid network compression visible as network thickness and volume decrease only in Arabidopsis after 50 min of light exposure compared with dark‐adapted plants (Fig. 1a,b,f). Meanwhile, TEM analysis showed a substantial decrease in the SRD parameter for both Arabidopsis and Ficus, with Arabidopsis showing a greater magnitude of change (*c*. 3 nm reduction in SRD; Fig. 1c,d,g).

To further investigate these structural changes, we employed SANS, which enables analysis of thylakoid ultrastructure – particularly SRD – with the unique advantage of being the nondestructive *in vivo* high‐resolution method, in contrast to TEM, which requires sample fixation. Based on detailed model fitting as described in Table S3, the SANS results (Fig. S2a) showed initially conflicting results compared with the TEM data for the illuminated samples. The main structural parameter from SANS is the fitted SRD value listed in Fig. S2(b). Results obtained using SANS showed that after 50 min of light, the Arabidopsis SRD expanded while the Ficus remained basically constant, in direct contradiction with the shrinkage measured with TEM.

This divergent result, obtained on the same species and under identical illumination conditions, was very interesting for us and indicated a greater complexity of the structural reorganization process than we initially anticipated. Therefore, we attempted to understand what could have caused the recorded discrepancies, in the context of both our own results and the literature data.

One of the potential hypotheses explaining registered variability could be related to plant growing conditions. Due to practical reasons, our Australia‐based SANS experiments were performed on plants grown in different conditions compared with those used in microscopy analyses performed in Europe. Specifically, Arabidopsis rosettes and Ficus plants used for microscopy dark–light experiments were grown in a glasshouse under naturally fluctuating light conditions, while for SANS measurements, Arabidopsis was grown in constant light conditions (12‐h photoperiod), and Ficus was obtained from a local supplier (flower shop) without glasshouse acclimatization. The influence of plant growing conditions on light‐induced structural changes will be further discussed in the last part of the Results section of this manuscript.

### Illumination time and grana structural dynamics: a meta‐analysis

**Fig. 2:**
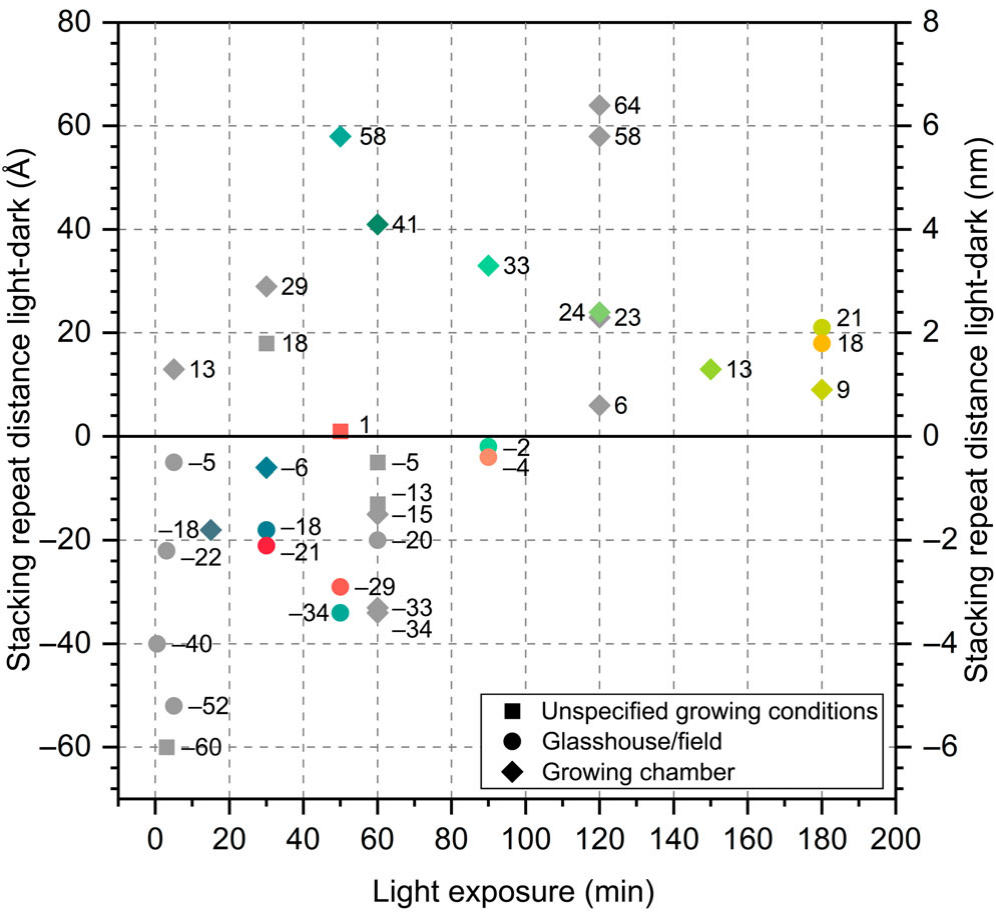
Change in stacking repeat distance of plant grana after subsequent minutes of light exposure. Gray points represent data from studies published during the last 50 yr (for details on organism, light intensity, and method used, see Supporting Information Table S2). Colorful points represent data obtained from this study using a color code consistent throughout the whole manuscript (blue, teal, green – data for Arabidopsis; red, orange, yellow – data for Ficus). Shapes of data points represent different growing conditions specified in the figure legend.

In light of conflicting data available in the literature and discrepancies in our results obtained using microscopy and scattering methods, we decided to conduct a meta‐analysis of published data, considering other factors that, beyond specific genotype or methodological artifacts, could account for the diversity of obtained results. Since not all details of plant growing and experimental conditions were provided in relevant publications, we decided to focus on illumination time first. In Fig. 2, we present changes in SRD values under light exposure in the context of illumination duration. Although the results are not entirely conclusive, suggesting the role of additional factors, such as intensity or the spectrum of the light used, the graph indicates a specific relationship between light exposure time and the direction and magnitude of SRD changes (gray data points on Fig. 2). Shorter exposure times were mainly related to SRD shrinkage, while longer exposure times were associated with SRD expansion. The meta‐analysis visualized in Fig. 2 served as our starting point for designing another experimental setup in which the structural response of thylakoids to illumination was linked to exposure time. The goal was to understand the dynamics of structural changes and address whether time is a critical factor in observing distinct structural changes across the results of different researchers.

### Light‐induced grana structural dynamics: a time‐course analysis

We continue to use our glasshouse‐grown Arabidopsis and Ficus plants. For this part of the study, we collected leaf samples before light exposure (after *c*. 16 h of darkness) and after exposure to intense light conditions for 30, 50, 90, and 180 min. From these time points, we obtained samples for TEM. Also, we isolated thylakoids for biochemical analyses to understand the mechanism underlying the observed structural dynamics of the granum vertical plane.

**Fig. 3:**
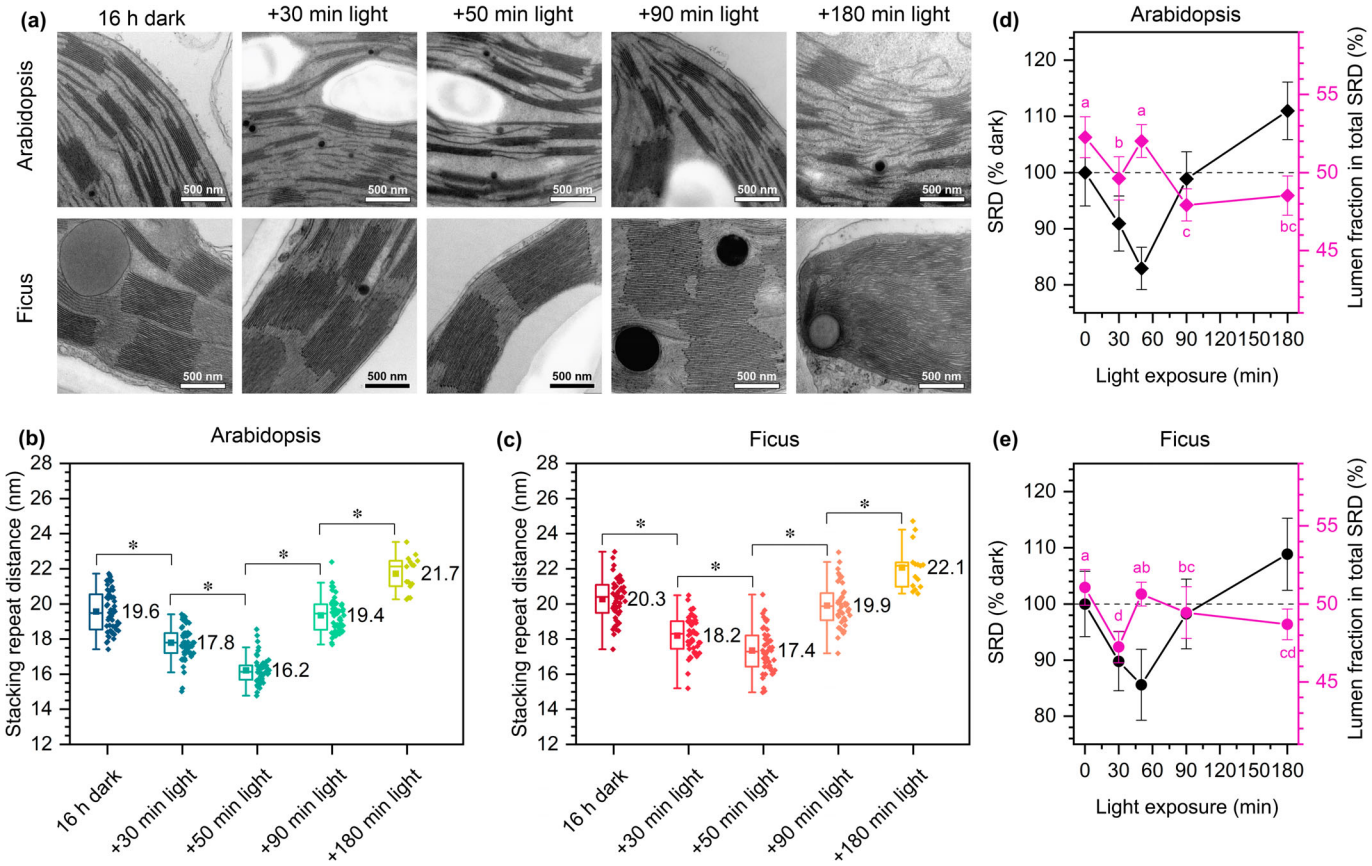
Time‐course analysis of light‐induced grana ultrastructural changes in glasshouse‐grown Arabidopsis and Ficus. (a) Electron micrographs of the thylakoid network in mesophyll chloroplasts of Arabidopsis and Ficus. (b, c) Quantitative analysis of stacking repeat distance (SRD) changes in Arabidopsis (b) and Ficus (c) during subsequent minutes of illumination. Transmission electron microscopy data were obtained from three biological replicates. The bottom and top of each box represent the 25 and 75 percentiles, respectively. The error bars denote SD; horizontal line and square mark the median and mean value, respectively; every point visible on the boxes represents an individual measurement. Pairs of results marked with an asterisk differ significantly at *P* ≤ 0.05; the comparison is presented for subsequent time points only (one‐way ANOVA with *post hoc* Tukey test; *n* = 15–55 (b), 15–53 (c)). (d, e) Quantitative analysis of the changes in the lumen fraction of the SRD in subsequent illumination time points (magenta). Results are presented in the context of total SRD values change (black) with respect to dark conditions. The error bars denote SD; pairs of results marked with different letters differ significantly at *P* ≤ 0.05 (one‐way ANOVA with the *post hoc* Tukey test; *n* = 10 (d, e)).

Quantitative analysis of TEM images revealed that both Arabidopsis and Ficus showed a decrease in the SRD value up to 50 min of light exposure, followed by an increase in subsequent time points, reaching values higher than those in leaves adapted to darkness (Fig. 3a–c). We enriched the analysis of SRD values by measuring the percentage of lumen fraction within the entire SRD using the Fast Fourier Transform (FFT) method (Fig. 3d,e). Changes in the lumen fraction over time were similar for both species. Our results indicate that during the initial phase of illumination, the change in SRD size primarily depends on a decrease in lumen height. After 50 min of illumination, when SRD size is at its lowest, the lumen‐to‐partition gap and membrane fraction suggest a uniform decrease in the height of both structural compartments. However, the subsequent increase in SRD size is mainly attributed to expanding the partition gap relative to the lumen size increase.

### Light‐induced biochemical changes in thylakoid membranes

**Fig. 4:**
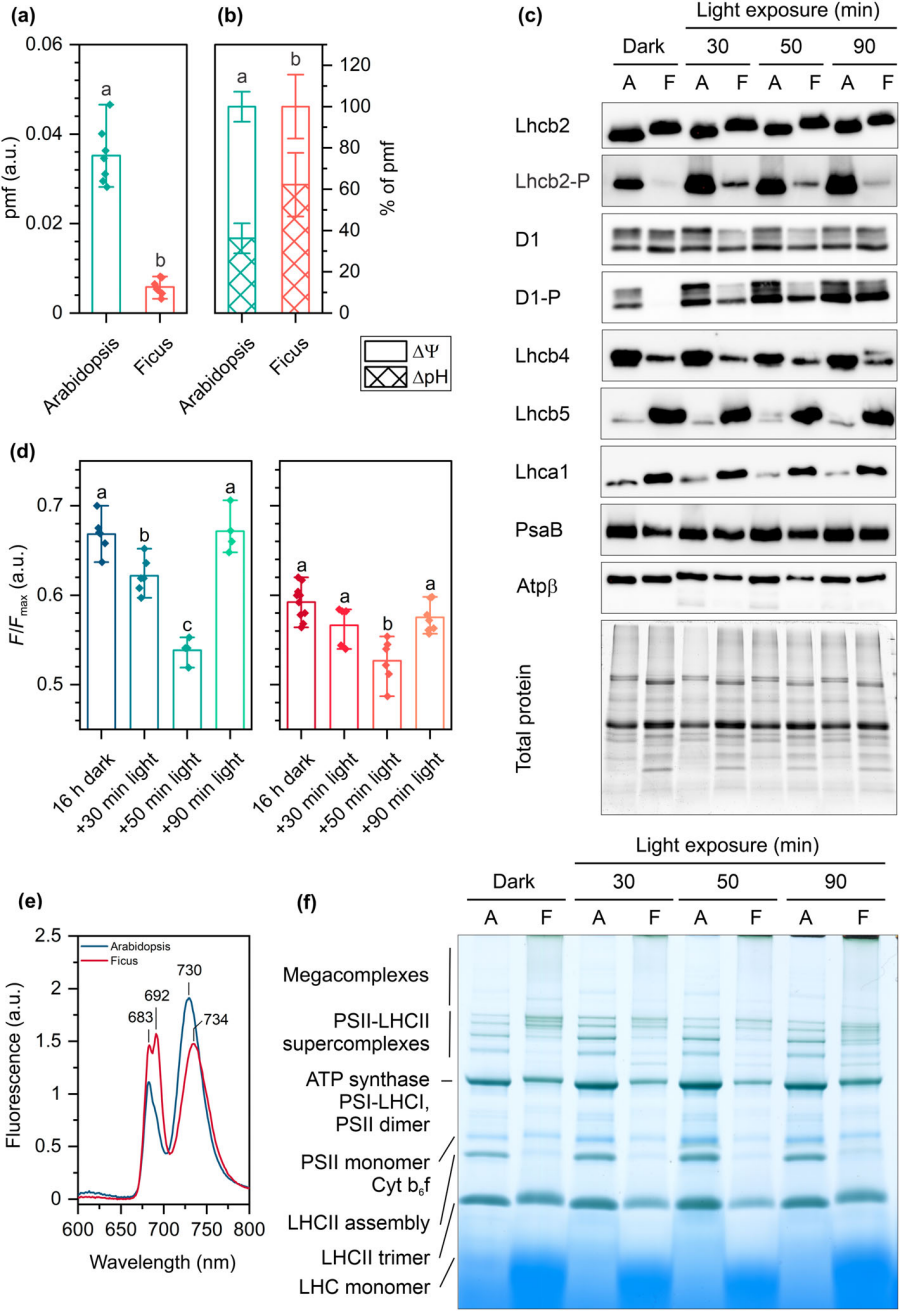
Spectroscopic and electrophoretic analyses of Arabidopsis and Ficus thylakoids isolated in subsequent time points of illumination. (a) Light‐induced proton motive force (pmf) estimated by the light–dark changes in the P515 signal; (b) Estimation of the components of pmf, transmembrane potential (Δψ), and proton gradient (ΔpH); (c) Immunoblots of selected Photosystem I (PSI) and Photosystem II (PSII) antennae (Lhca1, Lhcb2, Lhcb4, and Lhcb5) and core proteins (D1 and PsaB), phosphorylated variants of D1 and Lhcb2 proteins and Atpβ subunit of ATP synthase (for details on antibodies used, see the Materials and Methods section; due to lack of specificity of Lhcb1 and P‐Lhcb1 antibodies in Ficus samples, data of this analysis are presented in Supporting Information Fig. S4 only); densitometric analysis in Fig. S4. (d) Estimation of the membrane negative charge by 9‐aminoacridine fluorescence, the lower the *F*/*F*_max_ value, the higher the negative charge (left panel – Arabidopsis, right panel – Ficus); (e) Representative low‐temperature (77 K) Chl fluorescence emission spectra normalized to equal area under the curve. (f) Blue native polyacrylamide gel electrophoresis (BN‐PAGE) of Chl–protein complexes. A, Arabidopsis; F, Ficus; LHC, light‐harvesting complex. All presented data are representative of at least three independent experiments. The error bars denote SD; pairs of results marked with different letters differ significantly at *P* ≤ 0.05 (one‐way ANOVA with the *post hoc* Tukey test; *n* = 7–8 (a, b), 4–10 (d)).

Our further experiments focused on studying biochemical changes related to potential alterations in the lumen height and partition gap. To address the observed changes in lumen size, we assayed the pmf using an *in vivo* absorption method measuring electrochromic shift (ECS) at the 50‐min time point, where maximal thylakoid membrane shrinkage was observed. By comparing the absolute pmf values for Arabidopsis and Ficus under light intensities of *c*. 450 μmol photons m^−2^ s^−1^, we observed a sevenfold higher pmf in Arabidopsis (Fig. 4a). This effect was also evident in measurements performed under increasing light intensities (Fig. S3). For the 50‐min time point, we also calculated the proportion of the two pmf components, ΔpH and Δψ. Arabidopsis, which exhibited more pronounced thylakoid membrane shrinkage than Ficus, showed a lower proportion of ΔpH in the pmf at this time point (Fig. 4b). However, there are known controversies regarding the use of slow ECS signals to calculate the distribution of pmf between ΔpH and Δψ (Johnson & Ruban, 2014; Wilson *et al*., 2021). Therefore, the observed differences in ΔpH/Δψ ratios between the two species should be interpreted cautiously, as they may not accurately reflect actual pmf distribution. These differences might instead be directly related to varying levels of qE (energy‐dependent NPQ) and zeaxanthin between the studied species, which can influence the ECS signal due to the partial overlap of ECS with the Δ535 and Δ505 signals, respectively (Johnson & Ruban, 2014; Wilson *et al*., 2021).

To examine the contribution of the stromal side of the grana thylakoid membranes, we focused on analyzing changes in thylakoid components known to be induced by light and associated with stacking changes. We began by investigating changes in the phosphorylation status of selected antennae and core proteins of PSII influencing the net charge of the membrane (Fig. 4c) (Bellafiore *et al*., 2005; Bonardi *et al*., 2005). Light induced an increase in the phosphorylation of Lhcb2 and D1 proteins in Arabidopsis, with maximal values recorded after 30 and 50 min of illumination, respectively. Similarly, such an effect was also observed in Ficus, with a shift in the maximal signal detected for p‐D1 after 90 min of illumination.

We further investigated another protein modification correlated with structural changes: acetylation (Koskela *et al*., 2018; Rantala *et al*., 2022). Immunodetection of acetylated lysine revealed a gradual increase in the abundance of this modification induced by light in Arabidopsis. Surprisingly, no signal was detected in Ficus for any of the analyzed time points (Fig. S4). This absence of detectable acetylation in Ficus could be attributed to limited antibody reactivity with Ficus proteins or might represent a species‐specific adaptation of plants resulting in a favorable PSII‐LHCII complex organization in this shade‐tolerant species.

Since protein modifications influence surface charge density, we used a spectroscopic method based on 9‐aminoacridine fluorescence to track changes in the total negative charge of the membrane. A lower *F*/*F*_max_ value indicates a higher amount of negative charge present on the membrane surface. We observed a similar pattern of changes in this parameter in Ficus and Arabidopsis, with more pronounced changes in Arabidopsis. Interestingly, the increase in negative charge was noted until 50 min of illumination (minimal SRD value), while after 90 min of illumination, values were similar to those observed for dark‐adapted leaves, despite the lack of a significant decrease in protein phosphorylation (Fig. 4d).

Changes in negative surface charge are also known to be dependent on the amount of LHCII proteins. However, we did not observe any significant changes in the amount of tested LHCIIs throughout the illumination process. Interestingly, we observed distinct patterns of Lhcb4 and Lhcb5 abundance between Ficus and Arabidopsis, which can explain the significantly different spectral properties of thylakoids in these species (Fig. 4c,e).

Given that the organization of protein complexes is also considered an important factor in thylakoid stacking, we performed BN‐PAGE analysis of photosynthetic complexes' organization at the examined time points (Fig. 4f). We identified species‐specific differences in the organization of photosynthetic complexes, confirming the observed differences in low‐temperature Chl fluorescence spectra for dark‐adapted plants (Fig. 4e,f). Particularly in Ficus, we observe more abundant PSII–LHCII supercomplexes of higher molecular mass compared with Arabidopsis. Tracking changes in complexes organization due to high light induction showed the most pronounced changes in the PSII–LHCII supercomplexes region in Ficus after 90 min of illumination compared with other studied time points. Ninety minutes of light resulted in the formation of a diverse pool of PSII–LHCII supercomplexes with similar abundance of each registered form (Fig. 4f).

### The impact of growing light conditions on grana structural dynamics

**Fig. 5:**
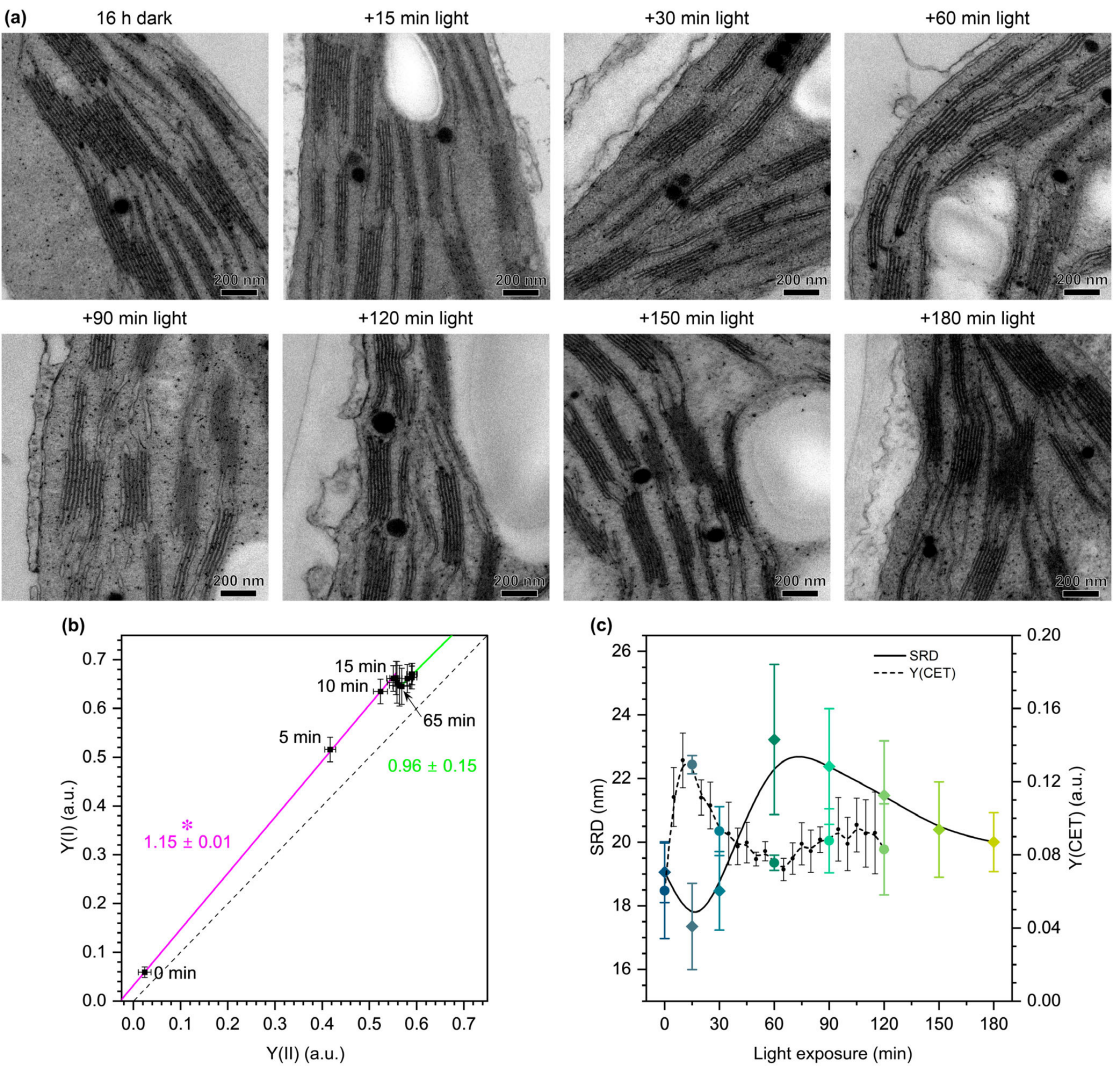
Time‐course analysis of light‐induced changes in thylakoid ultrastructure and cyclic electron transport efficiency (Y(CET)) of Arabidopsis plants grown in control light conditions. (a) Representative electron micrographs of thylakoid network in mesophyll chloroplasts of Arabidopsis. (b) The relationship between estimated photochemical efficiencies of Photosystem I (Y(I)) and Photosystem II (Y(II)); each point represents different illumination times. For clarity, only every third point is shown for the 20–120 min time. Colored lines and text represent the linear fit and its slope for 0–15 min (magenta) and 65–120 min (green) time points; the asterisk indicates the significant difference (*t*‐test, *P* ≤ 0.05) in the slope when compared to 1 (dashed line indicates Y(I) = Y(II)). (c) Quantitative analysis of stacking repeat distance (SRD) and Y(CET) changes in subsequently analyzed time‐points of illumination; note that Y(CET) was calculated as a difference between Y(I) and Y(II) in each analyzed time point. Transmission electron microscopy data were obtained from three biological replicates. Data points indicate mean value with error bars denoting SE (b) or SD (c).

Finally, we wanted to address the most pronounced discrepancy between our TEM results showing time‐related changes of initial shrinking followed by further thylakoid expansion and literature data. In our meta‐analysis, we observed that in some cases, SRD increase is observed already within the first 30 min of illumination (Kirchhoff *et al*., 2011; Clausen *et al*., 2014; Li *et al*., 2020), which was significantly earlier than in other analyses (Fig. 2). We compared our growing and illumination conditions with conditions reported in these studies, and the common registered difference was related to growing light conditions. In our case, these were natural light conditions of the glasshouse with daily fluctuating intensity due to, for example, changing cloud cover, in contrast to constant light of 120–150 μmol photons m^−2^ s^−1^ used in the mentioned reports. Therefore, we grew Arabidopsis plants in 150 μmol photons m^−2^ s^−1^ in a day/night cycle and collected TEM samples using an even more time‐dense protocol with 15, 30, 60, 90, 120, 150, and 180 min of 450 μmol photons m^−2^ s^−1^ illumination. We observed a similar pattern of structural changes, but the dynamics of these fluctuations were significantly altered. A minimal SRD value was registered after 15 min of illumination, followed by a dynamic increase leading to maximal SRD reached after 60 min of light exposure. This value was significantly larger than for dark‐adapted plants. However, further minutes of illumination resulted in a gradual decrease in SRD, reaching values similar to those of dark‐adapted plants by the end of the experiment (Fig. 5; for full statistics, see Fig. S5). As such, we showed that stable light‐growing conditions can lead to a more dynamic response of the thylakoid structure to light illumination; however, the shrinkage‐followed‐by‐expansion scheme is preserved in both cases, and we conjecture this is a general phenomenon. Such a hypothesis is in line with our SANS experiment in which for Arabidopsis grown in constant light conditions of *c*. 125 μmol photons m^−2^ s^−1^, the significant thylakoid expansion was registered already after 50 min of illumination (Fig. 2).

For the Arabidopsis growth chamber setup, we finally measured also the yields of PSI and PSII in subsequent minutes of *c*. 450 μmol photons m^−2^ s^−1^ illumination. Measurements were conducted at 5‐min intervals for 120 min of leaf illumination, coinciding with the time point at which SRD values observed in TEM studies stabilized (Fig. 5). Throughout the entire measurement time, we consistently recorded higher Y(I) than Y(II) efficiency (Fig. 5b). However, during the initial stage (0–15 min), which corresponded with thylakoid membrane shrinking, we observed a faster increase in Y(I) relative to Y(II) (slope 1.15 ± 0.01), suggesting higher CET efficiency than LET. In subsequent measurement points (20–65 min), we observed a decrease in Y(I) values with a simultaneous increase in Y(II) values. From 65 min onward, when SRD slowly began decreasing to values recorded in darkness, we observed a uniform increase in both Y(I) and Y(II) parameters (slope 0.96 ± 0.15), indicating a balance between both electron transport pathways (Fig. 5b,c).

We also used measured Y(I) and Y(II) values to plot the CET efficiency (Y(CET)) as Y(I)‐Y(II), which enabled direct comparison of Y(CET) with SRD value changes on a timescale (Fig. 5c). This approach was particularly valuable since the electron transport chain is considered a primary target of regulation through grana structural changes in the vertical plane (see the Introduction section). Higher Y(CET) values calculated using this method are considered to indicate a greater contribution of CET to total electron transport in thylakoid membranes (Huang *et al*., 2010). We recorded an increasing CET efficiency up to 15 min of illumination (maximal thylakoid shrinking), followed by a dynamic decrease to minimal values after 65 min of illumination, when thylakoids were fully expanded. In subsequent time points, we observed slight fluctuations in CET efficiency. However, despite the gradual decrease in SRD to levels comparable to dark conditions registered after 120 min of illumination, we did not detect any further dynamic CET regulation directly associated with structural changes (Figs 3b, S5).

## Discussion

The results presented in this work indicate that light‐induced structural modifications of grana stacks in their vertical plane cannot be considered a simple binary model in which we can differentiate only two morphological states related to dark and light conditions. We showed that bidirectional structural changes are mainly associated with the light exposure time and might be modulated by the prior growing conditions. This conclusion is based on studies presented here and a meta‐analysis of data published during the last 50 yr, indicating an overlooked phenomenon. As such, it suggests that the lately proposed models describing mechanisms behind and explaining the role of light‐induced thylakoid expansion (Puthiyaveetil *et al*., 2017; Gu *et al*., 2022, 2023) need refinement, considering that temporal shrinkage precedes thylakoid expansion, which is then followed by the relaxation of the stretched state to values comparable to those noted for dark‐exposed plants. Such behavior of an initial damped and then amplified oscillation is a trademark of responsive systems found widely in biology due to regulatory delays or feedback amplification, and particularly where there are transitions between different regimes of dynamics (Keener & Sneyd, 2009) as we have precisely suggested here for the regulation of CET and LET. We can, in principle, fit our structural relaxation data with an *ad hoc* model combining various terms of exponentially damped oscillations, but we leave it to future work to establish a more predictive model based on first principles to describe the clearly correlated behavior between the electron transfer and the mesoscopic membrane remodelings.

The complete pattern of structural changes (shrink‐expand‐relax) over 180 min light exposure was observed only in plants growing under constant light conditions, while glasshouse plants showed stretched dynamics (Fig. S6). Further time points could not be assessed in glasshouse plants due to starch accumulation affecting analysis reliability.

In this work, we started with a commonly used approach of analyzing structural changes in two states – dark and light conditions, but utilizing three parallel methods (CLSM, TEM, and SANS) to primarily better understand literature discrepancies and select a method for further analysis. We acknowledge the methodological challenges of combining CLSM (3D) with TEM (2D) imaging approaches. These concerns are particularly relevant when extracting volume information, as highlighted by recent studies (Harwood *et al*., 2020; Knoblauch *et al*., 2024). As such, we deliberately avoided direct comparisons between CLSM and TEM data at the same structural level and primarily used SRD measurements from TEM, which are less affected by dimensional interpretation issues when perpendicular grana cross‐sections are analyzed. Our CLSM measurements, despite resolution limitations, confirmed the direction of structural changes observed with TEM, revealing a substantial decrease in whole thylakoid network thickness and volume in Arabidopsis upon illumination – a global contraction that most probably extends beyond the SRD changes, also capturing a possible decrease in the distances between neighboring grana stacks in the vertical plane visible only *in vivo*. For reliable interpretation of SANS data, we used a complete mathematical model for thylakoid structure analysis initially proposed by us to analyze cyanobacterial membranes (Jakubauskas *et al*., 2019) and adapted for the plant thylakoids here. The model proved to be able to describe the data although with a number of assumptions and molecular constraints. Nevertheless, in light of the complex dynamics we reveal in this work, it is not surprising that the derived parameters are not in complete agreement with the TEM data. The latter more closely represents discrete structural snapshot in time compared to the SANS measurements, which collected data over 50‐min periods and thus averaged across the full range of dynamics document in Fig. 5, and the output from the scattering analysis reflects that. The dark‐state measurements should, however, be comparable and are also closer to the TEM data, although slightly larger. As discussed previously, there is an inherent difference between size distributions obtained via microscopy (which are number‐weighted) and scattering methods (volume‐weighted) (Jakubauskas *et al*., 2019). The latter typically produces slightly larger numbers consistent with our data.

Thus, the microscopy and scattering methods gave conflicting results, which, together with conflicting literature data, suggested to us that other factors, such as experimental setup artifacts, were important, and therefore, we focused our attention on the timescale of light exposure. However, it should be noted that light intensity, color, and other environmental factors, such as humidity and temperature, can probably also influence the dynamics of the observed structural changes. Still, their reliable assessment based on already published data was not possible due to the limited information provided by the authors, nor were they a subject of this study.

Interestingly, it was also proposed that different structural outcomes of illumination might be directly related to the object of observation, that is to the grana size (Gu *et al*., 2022). To address this hypothesis, we simultaneously studied Arabidopsis exhibiting typical‐sized grana stacks and Ficus characterized by giant grana structures. The TEM results showed an identical pattern of structural changes with only a smaller magnitude observed in Ficus. It should be noted that while our detailed time‐course experiments were conducted exclusively on C3 dicot plants, our meta‐analysis included studies on both C3 and C4 plants, including data from maize (C4) and barley (C3 monocot) as documented in Tables S1 and S2. Although our meta‐analysis suggests similar general patterns across plant types, the specific dynamics of thylakoid structural changes might vary in monocots and C4 plants, which would require dedicated studies to confirm whether the temporal patterns we observe are universally applicable across diverse photosynthetic strategies.

When analyzing literature data, it is visible that values recorded for particular species, growing and illumination conditions, applied visualization, and fixation methods can lead to nominally different results (Tables S1, S2). For instance, even within this study, we observed different SRD values for dark‐adapted Arabidopsis depending on the time of dark exposure, with values varying between *c*. 18 and 19 nm on average in plants darkened for 48 h vs 16 h, respectively. Moreover, the SRD value of Arabidopsis noted for the dark conditions recorded lately by Li *et al*. (2020) for HPF‐FS and microwave/chemical fixation was *c*. 17 nm, indicating that some variability might also be related to the fixation procedure. However, all noted SRD values are within the limits calculated from membrane and complex sizes in the vertical plane, limiting the minimal size of the lumen and partition gap (Kirchhoff *et al*., 2011; Albanese *et al*., 2017).

Given that light induces rapid structural changes within minutes, sample collection and fixation required careful handling in darkness or dim green photosynthetically inactive light, which is not easy to achieve in typical setups used for HPF fixation and *in vivo* measurements in scattering studies. We used dim green light conditions throughout ‘dark’ sample collection, fixation, and measurement.

For several years now, a quite extensive debate supported by *in silico* modeling has been taking place, centered around understanding the mechanisms behind observed size changes in SRD and its structural components (lumen, membrane, and partition gap). However, those studies consider just two, dark vs light, structural states of thylakoids, focusing on light‐induced lumen expansion presented recently (Kirchhoff *et al*., 2011; Li *et al*., 2020).

To understand the functional and biochemical basis of observed structural changes, we performed a set of experiments considering membrane components and processes that have been indicated as crucial to light‐induced thylakoid reorganization in the vertical plane (see references in the Introduction section). These were, in particular, changes in: membrane negative charge density; thylakoid protein modifications (phosphorylation and acetylation); reorganization of Chl–protein complexes, all associated with the interactions in the partition gap space; and pmf build‐up, related to lumen size changes. The listed set of experiments does not cover all the literature‐discussed factors, such as changes in chloroplast osmolarity, Cl^−^, K^+^, Mg^2+^ ion concentration, or other known thylakoid protein modifications (methylation and polyamination) (Ioannidis *et al*., 2012; Alban *et al*., 2014; Kunz *et al*., 2024), difficult to obtain in studies focused on the duration of light exposure. Our biochemical results in a time‐course setup did not provide a clear picture, which could associate observed structural dynamics with molecular changes suggested by earlier *in silico* and experimental studies obtained for binary dark vs light setups. For instance, it was shown previously that a low level of protein phosphorylation in the darkness, influencing the total negative surface charge of the thylakoid membranes, should result in increased membrane stacking due to lower repulsive forces between neighboring membranes in the stack (Puthiyaveetil *et al*., 2017). However, although we observed light‐induced protein phosphorylation maintained throughout the whole analyzed period, changes in the size of the partition gap were bidirectional, with initial shrinking followed by further expansion. Experimental data of other groups also confirm that the decrease in the partition gap size can coincide with the light conditions promoting PSII and LHCII phosphorylation. Li *et al*. (2020) showed that partition gap size decreases after light illumination (4.8–4.2 nm) of Arabidopsis leaves. A similar effect was observed in *in vitro* studies of Janik *et al*. (2013), where distances between membrane layers formed by lipid‐LHCII assemblies were smaller for a lipid‐P‐LHCII variant of the mixture. Moreover, in our measurements of the total negative thylakoid membrane charge, we did not observe the direct correlation between obtained values and the level of protein phosphorylation. Although up to 50 min of light negative surface charge increases in both species, the 90 min timepoint exhibits values similar to those recorded for dark plants. The nature of such dynamics is not known, particularly because no simultaneous changes in LHCII amounts were observed. Due to the methodology used for the total negative surface charge assessment, which does not discriminate between the membrane side, we can speculate that the observed decrease in negative charge after 90 min of illumination might result from not‐yet‐identified changes at the lumenal side of the membrane rather than on the well‐characterized stromal side. It is worth noticing that 90 min of illumination was the last time point analyzed from a biochemical perspective, since thylakoid samples collected after longer light exposure were characterized by high starch content, which resulted in poor quality of the obtained electrophoretic data. However, 90 min of illumination was enough to register significant alterations in PSII–LHCII supercomplex abundance in Ficus, suggesting that the regulation of the photosynthetic efficiency via massive complexes reorganization succeeds the dynamic control of grana thylakoid structure in their vertical plane. It should be acknowledged that all biochemical measurements were performed on isolated thylakoids, where the isolation procedure takes *c*. 1 h, which could potentially reverse or alter dynamic light effects, mimicking processes that occur in living plants. As such, these results should be interpreted with caution.

Pmf *in vivo* measurements revealed lower values for Ficus with its giant grana than for Arabidopsis, consistent with literature data showing the correlation between grana size and pmf values, as demonstrated by Ioannidis *et al*. (2012) in tobacco plants with elevated LHCII polyamination. Moreover, literature data suggest a correlation between increased ΔpH values and water influx into the lumen caused by counter ion fluxes (Kirchhoff *et al*., 2011). Our results indicate a lower percentage of ΔpH in Arabidopsis than in Ficus. Both the higher total values achieved for Arabidopsis and the potentially low ΔpH proportion in Arabidopsis after 50 min of illumination (maximal lumen shrinkage) may explain the higher dynamics of structural changes within the lumen observed in this species.

Understanding the relationship between lumen size changes, pmf components (ΔpH and Δψ), and ion fluxes requires further research. While recent studies show fluctuations in ion transporter activity within minutes of light activation (Li *et al*., 2021), longer term dynamics and their relationship to lumen size changes remain to be explored using emerging tools for ion content measurements (Sadoine *et al*., 2023).

Fluctuations in lumen size have recently been identified as one of the key multifunction electron transport control/photoprotection mechanisms, alongside well‐studied mechanisms, such as NPQ and plastoquinol oxidation control (Havaux, 2020; Ruban & Wilson, 2020). Specifically, Gu *et al*. (2022) highlight the significant role of changes in lumen size in controlling the transport of PC between the lumen areas associated with grana thylakoids and those present in stroma thylakoids. This theory, called *ultrastructural control*, is supported by the latest simulations, which indicate that the magnitude of lumen swelling is sufficiently large to facilitate electron transport substantially (Höhner *et al*., 2020). On the other hand, lumen shrinking will result in macromolecular blocking and increase the likelihood of collisions between PC molecules, as well as between them and membrane protein domains protruding into the lumen region (Kirchhoff *et al*., 2011). A shrunken lumen can slow the electron transfer from PSII to PSI. This situation can be beneficial in the early stages of illumination when the Calvin–Benson–Bassham cycle is not fully active and when other regulatory mechanisms are not efficient enough to regulate electron transport within the required range. Particularly to protect PSI from photoinhibition associated with reactive oxygen species production and leading to damage of this complex, whose repair mechanisms are significantly slower and less efficient than those of PSII (Lima‐Melo *et al*., 2021; Su *et al*., 2023; Lee & Kim, 2024).

**Fig. 6:**
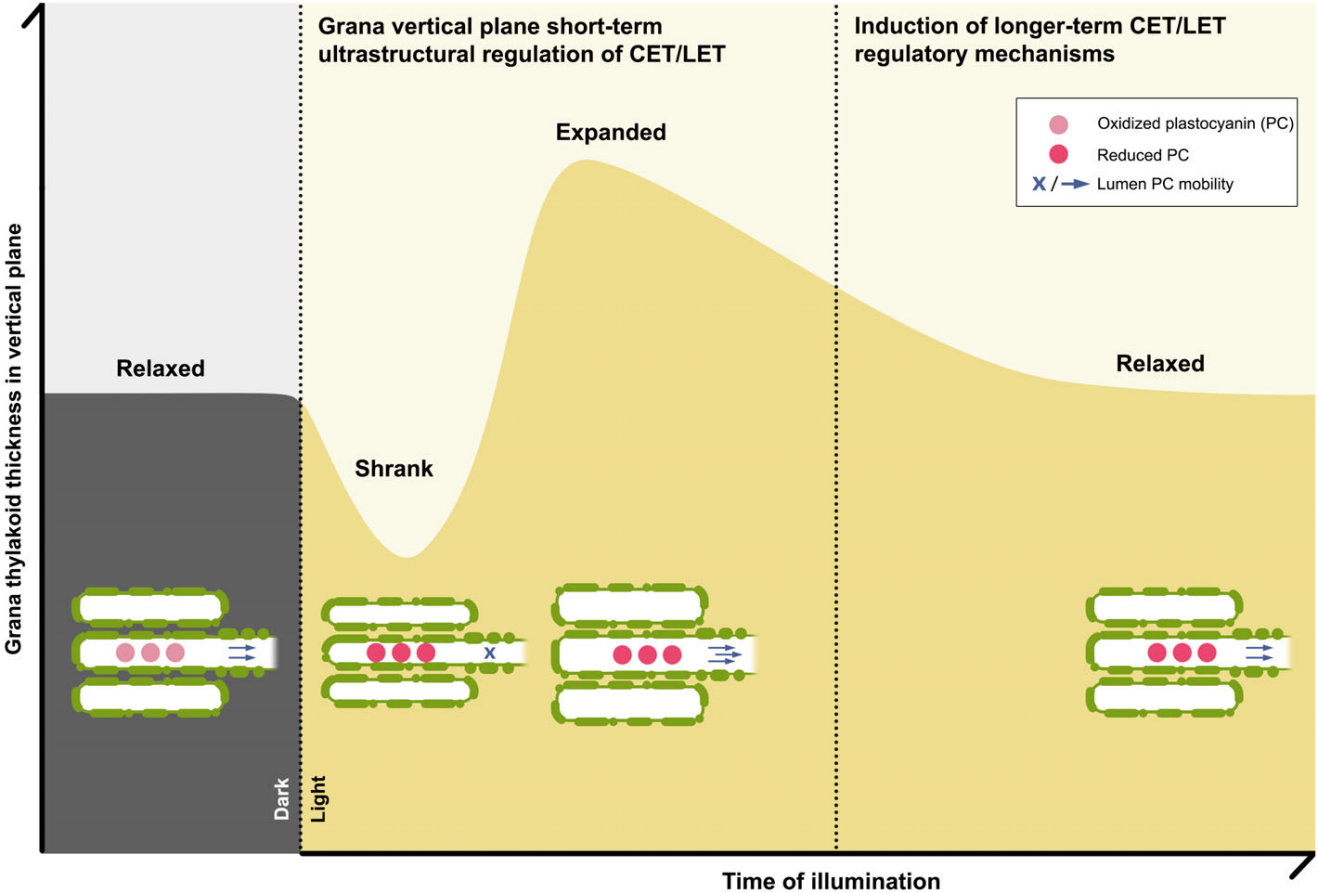
Scheme showing the relationship between grana vertical plane ultrastructural dynamics and plastocyanin (PC) diffusion efficiency influencing the cyclic to linear electron transport (CET : LET) ratio. Light triggers the electron transport chain and proton motive force build‐up, which is accompanied by grana ultrastructural changes fine‐tuning the balance between CET and LET by providing a steric hindrance for the PC diffusion in the shrank state and promoting its transport in the swollen lumen in the following expanded state. The subsequent relaxation phase is not limiting LET efficiency, suggesting the induction of other photosynthesis regulatory mechanisms.

To understand the dynamics of CET and LET efficiency regulation in our time‐course experimental setup, we conducted *in vivo* Chl *a* fluorescence measurements, which allowed us to calculate CET efficiency at successive stages of Arabidopsis plants illumination (growth chamber setup). For analyzing *in vivo* Y(CET) in direct comparison with structural parameters in our time‐course approach (crucial for our study), we employed a method despite its known limitations. Specifically, it should be noted that the difference between Y(I) and Y(II) values provides a reliable estimation of Y(CET), assuming the absence of alternative electron acceptors and pathways (Baker *et al*., 2007). To further confirm the validity of our approach, we also plotted the mutual dependence of Y(I) and Y(II) as the most commonly used method to establish the *in vivo* CET/LET relationship, and we obtained consistent results with respect to Y(I)–Y(II) analyses. The increase in CET efficiency correlates with the observed decrease in SRD in the early stages of light exposure, and the subsequent dynamic decrease in CET efficiency coincides with the maximum recorded SRD values after 60 min of illumination. In the later stages, as SRD gradually decreases again, approaching values characteristic of darkened samples, CET efficiency slightly increases. In the final measurement point, there is again a tendency toward a decrease in CET efficiency in favor of LET; however, it is no longer associated with changes in SRD values. This suggests the activation of other regulatory mechanisms that operate in the later stages of light exposure (Fig. 6). Among them, the reorganization of photosynthetic complexes or general changes in thylakoid structure, particularly the formation of thylakoid doublets significantly decreasing the PC diffusion distance (Garty *et al*., 2024) are worth mentioning. These mechanisms promote LET sufficiently without the need to maintain the system in a structurally stretched state.

To summarize, light‐induced grana dynamics in the vertical plane proceeds through three distinct stages: shrinkage, expansion, and relaxation to the dark‐registered state (Fig. 6). The biochemical mechanisms underlying these structural changes are more complex than previously recognized. Notably, plants growing in constant light conditions showed more rapid responses than those in glasshouse conditions (Fig. S6).

From a functional point of view, we demonstrated that an initial phase of thylakoid membrane shrinkage upon dark‐to‐light transitions is essential for effectively regulating the CET/LET ratio by blocking PC transport (Fig. 6). Our findings suggest that the dark‐adapted state of thylakoids can be considered a structurally relaxed state close to physical equilibrium. The membrane system strives to return to this relaxed state following an initial regulatory phase (dynamic shrinking followed by expansion), typically lasting minutes to a few hours. During this phase, other photosynthetic regulatory mechanisms become fully operational, ensuring optimal efficiency of the photosynthetic apparatus in subsequent hours of light exposure.

## Competing interests

None declared.

## Author contributions

JJKK and ŁK conceived the study. JW, RM, DJ, PEJ, KM, JJKK and ŁK planned and designed the research. JW, RM, DJ, AS, CG and ŁK performed experiments; JW, RM, DJ, AS, CG, JJKK and ŁK analyzed data. ŁK provided conception of the manuscript. ŁK wrote the article with a contribution of RM and JJKK. JW and RM contributed equally to this work.

## Disclaimer

The New Phytologist Foundation remains neutral with regard to jurisdictional claims in maps and in any institutional affiliations.

## Supporting information


**Fig. S1** Structural analysis of plastids and their membranes in leaf blades of shade‐tolerant variegated plants.
**Fig. S2** Ultrastructural parameters of Arabidopsis and Ficus thylakoid network obtained from small‐angle neutron scattering (SANS) analysis.
**Fig. S3** Light–dark transition‐induced changes of the P515 signal in Arabidopsis and Ficus plants.
**Fig. S4** Immunoblot quantitative analysis of selected thylakoid proteins.
**Fig. S5** Statistical analysis of the stacking repeat distance (SRD) and cyclic electron transport efficiency (Y(CET)) data for Arabidopsis plants.
**Fig. S6** Time‐course stacking repeat distance changes upon high light illumination in Arabidopsis and Ficus plants adapted to different growing light conditions.
**Methods S1** Detailed description of the small‐angle neutron scattering (SANS) modeling and membrane negative charge measurements.
**Table S1** Summary of experimental setups and structural outcomes in grana nanomorphology upon illumination.
**Table S2** Summary of quantitative stacking repeat distance (SRD) changes of various organisms in darkness and after illumination in given conditions.
**Table S3** Structural thylakoid membrane parameters for Arabidopsis and Ficus obtained from small‐angle neutron scattering (SANS) fitting.
Please note: Wiley is not responsible for the content or functionality of any Supporting Information supplied by the authors. Any queries (other than missing material) should be directed to the *New Phytologist* Central Office.

## Data Availability

The data that support the findings of this study are openly available in Dane Badawcze UW repository at doi: 10.58132/UZI5C7 reference number.
