# Supplementary material for: Shrink or expand? Just relax! Bidirectional grana structural dynamics as early light‐induced regulator of photosynthesis
Source: New Phytol. 2025 Apr 27;246(6):2580–96. doi: 10.1111/nph.70175 (PMC12095992; doi:10.1111/nph.70175)
Supplement: Supplementary file 1 — Fig. S1 Structural analysis of plastids and their membranes in leaf blades of shade‐tolerant variegated plants. Fig. S2 Ultrastructural parameters of Arabidopsis and Ficus thylakoid network obtained from small‐angle neutron scattering (SANS) analysis. Fig. S3 Light–dark transition‐induced changes of the P515 signal in Arabidopsis and Ficus plants. Fig. S4 Immunoblot quantitative analysis of selected thylakoid proteins. Fig. S5 Statistical analysis of the stacking repeat distance (SRD) and cyclic electron transport efficiency (Y(CET)) data for Arabidopsis plants. Fig. S6 Time‐course stacking repeat distance changes upon high light illumination in Arabidopsis and Ficus plants adapted to different growing light conditions. Methods S1 Detailed description of the small‐angle neutron scattering (SANS) modeling and membrane negative charge measurements. Table S1 Summary of experimental setups and structural outcomes in grana nanomorphology upon illumination. Table S2 Summary of quantitative stacking repeat distance (SRD) changes of various organisms in darkness and after illumination in given conditions. Table S3 Structural thylakoid membrane parameters for Arabidopsis and Ficus obtained from small‐angle neutron scattering (SANS) fitting. Please note: Wiley is not responsible for the content or functionality of any Supporting Information supplied by the authors. Any queries (other than missing material) should be directed to the New Phytologist Central Office. [file NPH-246-2580-s001.pdf]

New Phytologist

New Phytologist Supporting Information

Article title: Shrink or Expand? Just Relax! Bidirectional Grana Structural Dynamics as Early Light-Induced Regulator of Photosynthesis

Authors: Joanna Wójtowicz, Radosław Mazur, Dainius Jakubauskas, Anna Sokolova, Christopher Garvey, Kell Mortensen, Poul Erik Jensen, Jacob JK Kirkensgaard, Łucja Kowalewska

Article acceptance date: 02 April 2025

The following Supporting Information is available for this article:

Methods S1 Detailed description of the Small Angle Neutron Scattering (SANS) modeling and membrane negative charge measurements.
Fig. S1 Structural analysis of plastids and their membranes in leaf blades of shade-tolerant variegated plants.
Fig. S2 Ultrastructural parameters of Arabidopsis and Ficus thylakoid network obtained from Small Angle Neutron Scattering (SANS) analysis.
Fig. S3 Light-dark transition-induced changes of the P515 signal in Arabidopsis and Ficus plants.
Fig. S4 Immunoblot quantitative analysis of selected thylakoid proteins.
Fig. S5 Statistical analysis of the stacking repeat distance (SRD) and cyclic electron transport efficiency (Y(CET)) data for Arabidopsis plants.
Fig. S6 Time-course stacking repeat distance changes upon high light illumination in Arabidopsis and Ficus plants adapted to different growing light conditions.
Table S1 Summary of experimental setups and structural outcomes in grana nanomorphology upon illumination.
Table S2 Summary of quantitative stacking repeat distance (SRD) changes of various organisms in darkness and after illumination in given conditions.
Table S3 Structural thylakoid membrane parameters for Arabidopsis and Ficus obtained from Small Angle Neutron Scattering (SANS) fitting.

Methods S1

Detailed description of the Small Angle Neutron Scattering (SANS) modeling and membrane negative charge measurements.

SANS modeling

We follow (Jakubauskas *et al.*, 2019) for the modeling of the double bilayer grana stack. However, given the main result in this paper, the SANS measurements are inherently problematic to interpret as they most likely are an average over the dynamics reported in Fig. 5 which adds to the already high complexity of a structurally polydisperse and heterogeneous living system. Also, the model depends on a high number of parameters which are not all known a priori. Nevertheless, with some assumptions and molecular constraints we can fit the model to the data and get reasonable numbers out which roughly correlate with the microscopy data. In the modeling we use scattering length density values calculated in (Jakubauskas *et al.*, 2021) assuming a membrane composition of 75% protein and 25% lipid. We constrain the bilayer thickness to 4 nm. Also, we fix the number of layers to 5 and the Caillé parameter describing bending fluctuations to 0.01 reflecting the stiff nature of the grana stack membranes. The main structural fitting parameters are thus the Stacking Repeat Distance (SRD) value and either the lumen or partition gap distance. The model allows to center the system around either and let the size of the other be derived from the SRD value. In Jakubauskas et al. (2019) the natural choice was to center around the cyanobacterial lumen space, but here we choose the opposite as the partition gap is small and expected to be relatively constant compared to the lumen height. We include a 10% polydispersity in the SRD value. All fitting parameters and optimized values are given in Table S3. All model fits are smeared with a 7% pinhole resolution function.

Membrane negative charge measurements

To determine negative charge density on thylakoid membranes fluorescence method based on 9-aminoacridine fluorescence was applied (Chow & Barber, 1980a; Chow & Barber, 1980b). Thylakoid samples (100 µg) were washed 3 times in 0.5 ml 10 mM Hepes-NaOH (pH 7.5) buffer containing 0.1 M sorbitol and 1 mM EDTA. After each wash, samples were centrifuged at 10,000 x g for 3 minutes at 4°C. The final pellet was resuspended in the washing buffer to a final Chl concentration of 1.5 mg/ml. Fluorescence measurements were conducted using a Shimadzu RF5301-PC fluorometer in a quartz cuvette with stirring. Excitation and emission wavelengths were set to 340 nm (4 nm slit), and 455 nm (1 nm slit), respectively.

The 9-aminoacridine was added to a 2 ml Hepes buffer (1 mM Hepes-NaOH, pH 7.6, 0.1 M sorbitol, 1 mM EDTA and 20 µM DCMU) to a final concentration of 20 µM. After signal stabilization, thylakoid membranes were added to a final Chl concentration of 10 µg/ml, resulting in the fluorescence drop (i.e. 9-aminoacridine cation interacting with the negatively charged membrane). After reaching the plateau, the 3 µl of 50 mM EDTA was added to the cuvette giving final EDTA concentration of 1.06 mM. Next, 1 M $MgCl_2$ was added to a final concentration of 20 mM, causing the release of 9-aminoacridine from the membrane and resulting in a fluorescence rise (see below for representative data). The obtained fluorescence traces were normalized to the maximal fluorescence signal (F_{max}) in the presence of thylakoids. Due to method limitations described elsewhere (Bérczi & Møller, 1993), we did not calculate the negative charge density of the membrane, instead we compared the F/F_{max} values for washed thylakoids.

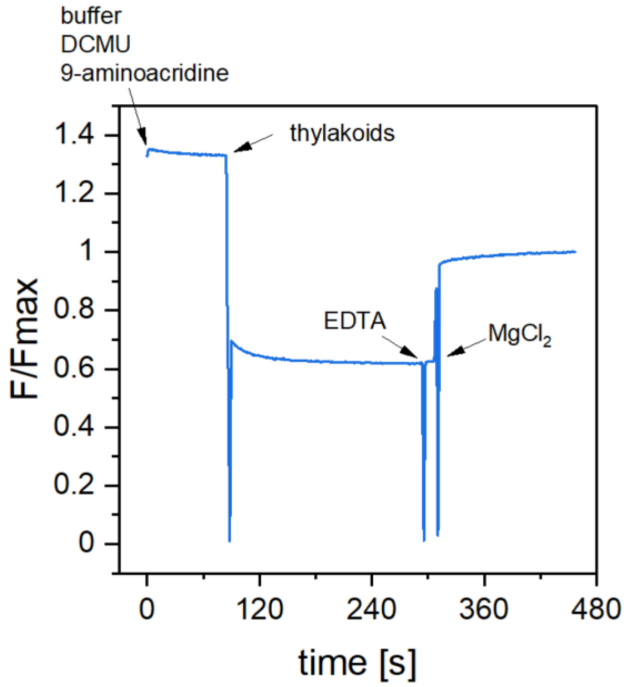

An example of 9-aminoacridine fluorescence changes during measurements for the estimation of the membrane negative charge. The signal was normalized to the maximal fluorescence in the presence of thylakoids. Arrows indicate time-points of adding reagents/samples.

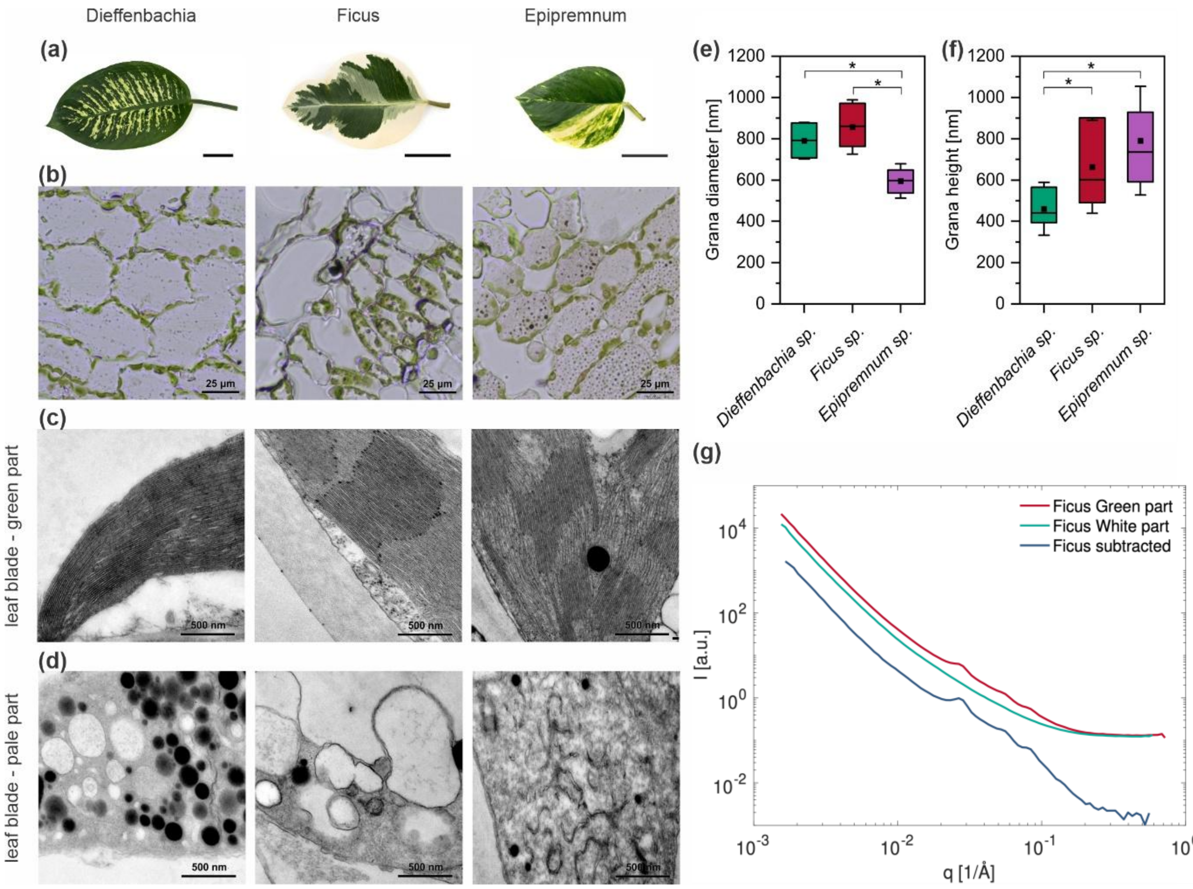

Fig. S1. Structural analysis of plastids and their membranes in leaf blades of shade-tolerant variegated plants. (a) - photographs of leaves of *Dieffenbachia seguine* (Jacq.) Schott, *Ficus elastica* Roxb. ex Hornem, and *Epipremnum aureum* (Linden & André) G.S.Bunting; note that data for respective species visualized using different methods are arranged in one column. (b) bright field light microscopy images of green parts leaf blade cross sections. (c) Transmission Electron Microscopy (TEM) images of chloroplast membrane network. (d) TEM images of plastid membrane network present in pale areas of leaf blade. (e) and (f) quantitative analysis of grana sizes based on TEM visualization (The bottom and top of each box represent 25 and 75 percentile, respectively. The error bars denote standard deviation (SD), horizontal line and square mark median and mean value, respectively. Pairs of results marked with asterisk differ significantly at $p \leq 0.05$ (one-way ANOVA with post hoc Tukey test; $n = 20$). (g) Example of background subtraction using the white part of Ficus.

(a)

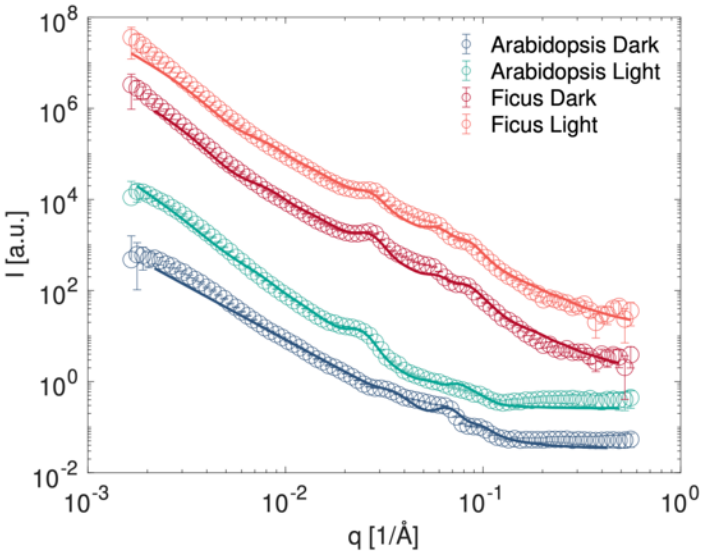

(b)

	SRD [nm]	Partition gap [nm]	Lumen width [nm]	Lumen [%]
Arabidopsis Dark	19.3	3.3	8.0	41.5
Arabidopsis Light	25.1	2.97	14.1	56.3
Ficus Dark	22.2	2.93	11.3	50.8
Ficus Light	22.1	3.43	10.7	48.3

Fig. S2. Ultrastructural parameters of Arabidopsis and Ficus thylakoid network obtained from Small Angle Neutron Scattering (SANS) analysis. (a) Scattering patterns and model fits for Arabidopsis and Ficus dark adapted and after illumination as described in Methods. (b) Main structural parameter from the SANS model fits assuming fixed 4 nm membrane thickness. Full list is provided in Table S3. SRD, Stacking Repeat Distance.

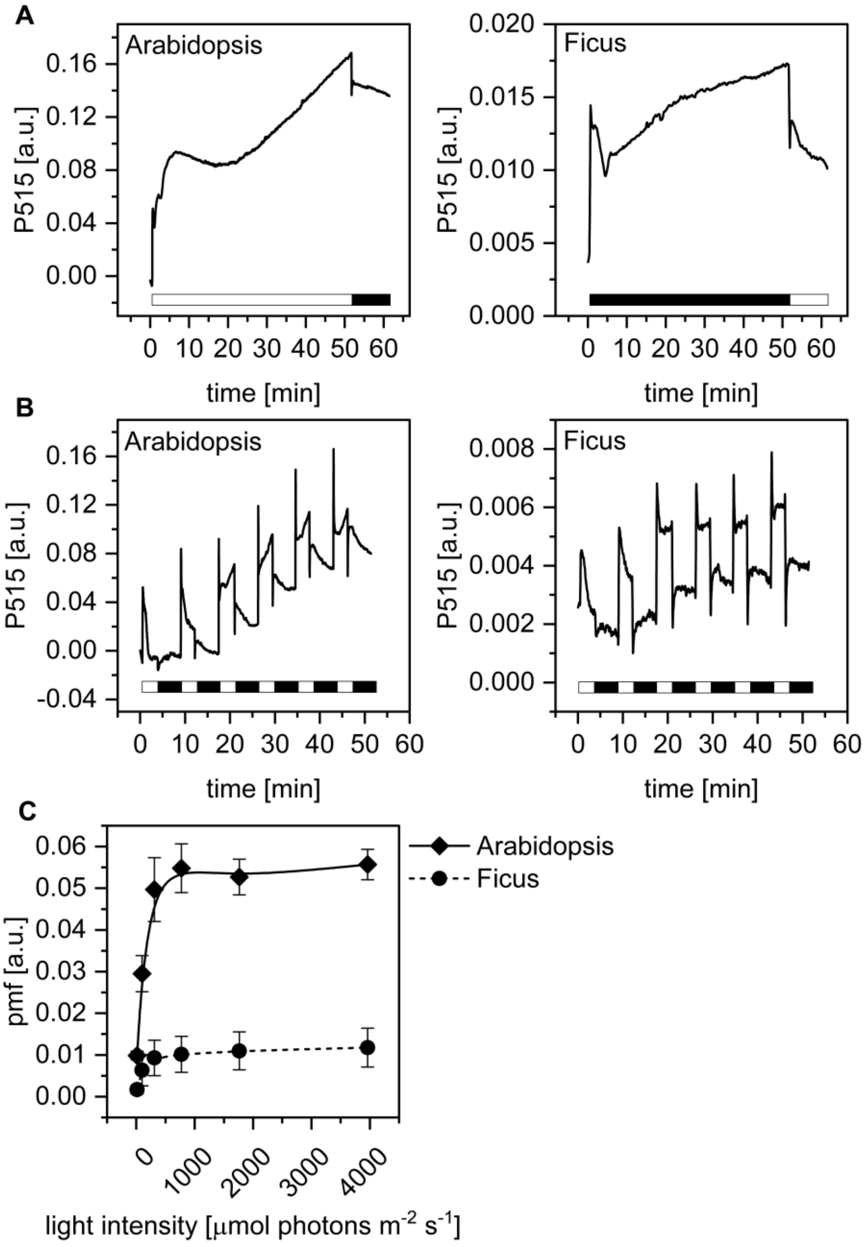

Fig. S3. Light-dark transition-induced changes of the P515 signal in Arabidopsis and Ficus plants. (A, B) representative traces of P515 signal for constant actinic light intensity (A) and for increasing actinic light intensity (B); white and black boxes represent the actinic light illumination and dark periods, respectively. (C) Light-induced proton motive force (pmf) estimated by the light-dark changes of the P515 signal under increasing actinic light intensity; The error bars denote standard deviation (*n* = 6; each leaf from different plant).

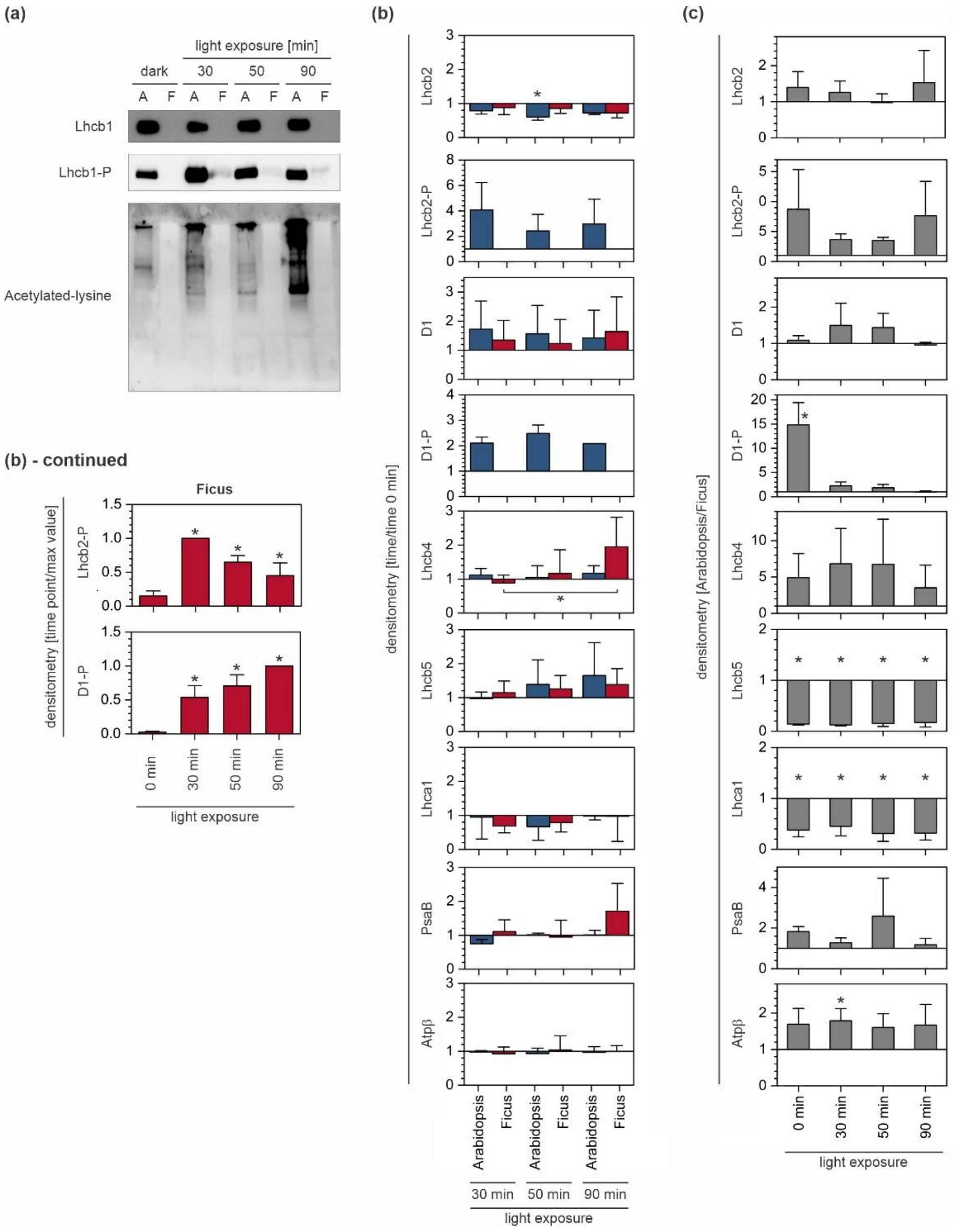

Fig. S4. Immunoblot quantitative analysis of selected thylakoid proteins. (a)immunoblot immunodetection of Lhcb1, P-Lhcb1 and acetylated-lysine in Arabidopsis and Ficus thylakoids (blots present results of a single experiment). (b) and (c) densitometric analysis for immunoblots presented in Fig. 4; note that densitometric analysis on panel (b) was normalized to the 0 min time-point except for Ficus Lhcb2-P and D1-P immunoblots where the normalization to the maximal value was used. The error bars denote standard deviation; pairs of results marked with an asterisk differ significantly at $p \leq 0.05$ (one-way ANOVA with post hoc Tukey test; n =2-4).

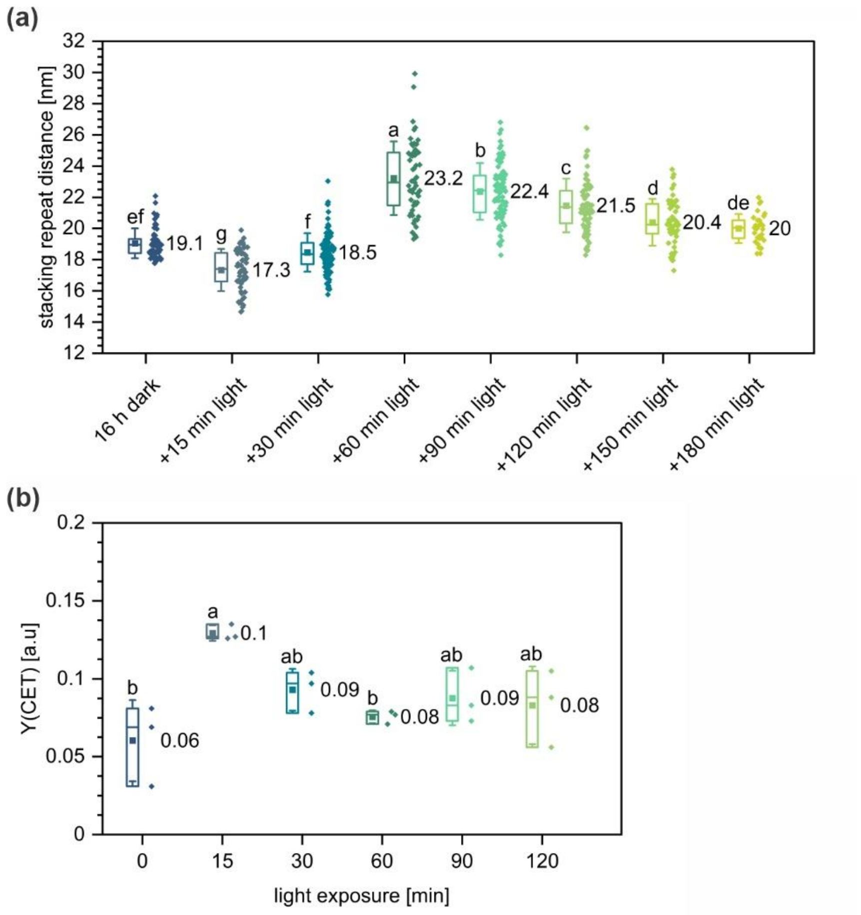

Fig. S5. Statistical analysis of the stacking repeat distance (SRD) and cyclic electron transport efficiency (Y(CET)) data for Arabidopsis plants. (a) SRD, (b) Y(CET). The bottom and top of each box represent 25 and 75 percentile, respectively. The error bars denote standard deviation (SD), horizontal line and square mark median and mean value, respectively. Pairs of results marked with different letters differ significantly at $p \leq 0.05$ (one-way ANOVA with post hoc Tukey test; n = 31-113 (panel a), 3 (panel b).

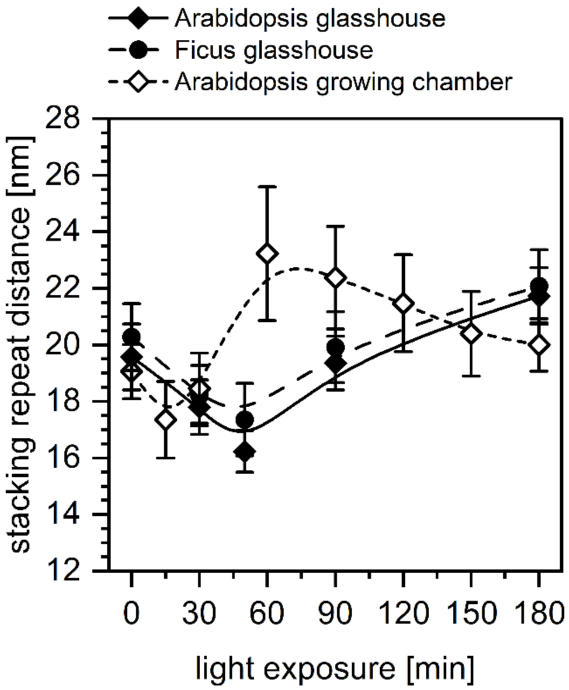

Fig. S6. Time-course stacking repeat distance changes upon high light illumination in Arabidopsis and Ficus plants adapted to different growing light conditions. The error bars denote standard deviation (data from Fig. 3 and 5).

Table S1. Summary of experimental setups and structural outcomes in grana nanomorphology upon illumination. HL - high light, NL - normal light, LL - low light; ChemF - chemically fixed samples analyzed in Room Temperature (RT) Transmission Electron Microscopy (TEM); HPF-FS - high pressure freezed and freeze substituted samples analyzed in RT TEM; digitonin fractionation - chlorophyll amount registered in isolated thylakoids fractionated using digitonin.

Organism	Entity	Effect of dark to light transition	Illumination	Light color, time	Method	Reference
Arabidopsis Col 0	Isolated thylakoids in buffer	Decrease in thylakoid stacking	500-1000 µmol photons m^{-2} s^{-1}	White, 20-60 min	Digitonin fractionation, light scattering	(Yamamoto *et al.*, 2008)
	Leaves	Increase in grana number per chloroplast	150 µmol photons m^{-2} s^{-1}	Growth light, 60 min	ChemF	(Anderson *et al.*, 2012)
	Leaves grown in LL	No significant changes in grana size	1000 µmol photons m^{-2} s^{-1}	White, 30 min	ChemF	(Schumann *et al.*, 2017)
	Leaves grown in NL	No significant changes in grana size	1000 µmol photons m^{-2} s^{-1}	White, 30 min	ChemF	(Schumann *et al.*, 2017)
	Leaves grown in HL	Decrease in grana stack height	1000 µmol photons m^{-2} s^{-1}	White, 30 min	ChemF	(Schumann *et al.*, 2017)
	Leaves grown in NL	Expansion of grana thylakoids	250-300 µmol photons m^{-2} s^{-1}	White, 30 min	HPF-FS, microwave ChemF	(Li *et al.*, 2020)
	Leaves grown in LL	Decrease in thylakoid number per grana stack; no significant changes in grana diameter	200 µmol photons m^{-2} s^{-1}	Orange, 60 min	ChemF	(Guardini *et al.*, 2022)
	Leaves grown in NL	Transition of the fraction of typical grana stacks to thylakoid doublets	100 µmol photons m^{-2} s^{-1}	White, 120 min	ChemF	(Garty *et al.*, 2024)

Barley	Leaves	Transition to typical grana/stroma organization from a swollen lumen state	130 µmol photons m^{-2} s^{-1}	White, 180 min	HPF-FS	(Pfeiffer & Krupinska, 2005)
Monstera	Leaves grown in HL	Expansion of grana thylakoids; decrease in thylakoid number per grana	1500 µmol photons m^{-2} s^{-1}	White, 20 min	ChemF	(Demmig-Adams *et al.*, 2015)
	Leaves grown in LL	No significant changes in grana size and periodicity	1500 µmol photons m^{-2} s^{-1}	White, 20 min	ChemF	(Demmig-Adams *et al.*, 2015)
Spinach	Attached leaves	Decrease in grana appression	300-1500 µmol photons m^{-2} s^{-1}	Sunlight, 30-180 min	ChemF	(Rozak *et al.*, 2002)
	Isolated thylakoids in buffer	Thylakoid unstacking under HL *in vitro*	500-2000 µmol photons m^{-2} s^{-1}	White, 10-60 min	Digitonin fractionation	(Khatoon *et al.*, 2009)
	Leaves grown in NL	Decrease in thylakoid number per grana; increase in grana number per chloroplast	350 µmol photons m^{-2} s^{-1}	White, 60 min	ChemF	(Wood *et al.*, 2019)

Table S2. Summary of quantitative stacking repeat distance (SRD) changes of various organisms in darkness and after illumination in given conditions. SANS – small-angle neutron scattering; ft-c – foot-candle, +ATP - with 1 mM ATP added; ChemF - chemically fixed samples analyzed in Room Temperature (RT) Transmission Electron Microscopy (TEM); HPF-FS - high pressure freezed and freeze substituted samples analyzed in RT TEM. Results included in the ranges in the Table are presented as points for the average value in Fig. 2; ± sign indicates standard deviation; for papers where the SRD values were not provided we calculated SRD based on published TEM micrographs.

Organism	Entity	SRD Dark, Å	SRD light, Å	Illumination	Light color, time	Method	Reference
Arabidopsis	De-enveloped chloroplasts in buffer	252±2	316±7 /+ATP<br>275±4 /-ATP	2000 µmol photons m^{-2} s^{-1}	White, 120 min	ChemF	(Chuartzman *et al.*, 2008)
	Leaf degassed with buffer	168±4	186±4	500 µmol photons m^{-2} s^{-1}	White, 30 min	HPF-FS	(Kirchhoff *et al.*, 2011)
	Isolated thylakoids in buffer	200±100	213±100	150 µmol photons m^{-2} s^{-1}	Red 640 nm, 5 min	HPF-FS	(Clausen *et al.*, 2014)
	Attached leaf	175	233±8	2000 µmol photons m^{-2} s^{-1}	White, 120 min	HPF-FS	(Tsabari *et al.*, 2015)[0]
	Leaves grown in "normal light"	170	199.7±1	250-300 µmol photons m^{-2} s^{-1}	White, 30 min	HPF-FS, microwave ChemF	(Li *et al.*, 2020)
	Leaves grown in "low light"	201±52	207±30	200 µmol photons m^{-2} s^{-1}	Orange, 60 min	ChemF	(Guardini *et al.*, 2022)
Maize	Leaf	101±7	88±8<br>96±8	1400 µmol photons cm^{-2} s^{-1}	Red (650±7.5 nm), 60 min<br>Far Red (707±8.5 nm), 60 min	ChemF	(Mustárdy *et al.*, 1976)
Pea	Leaf	195±4	from 152±4 to 172±3	1-37 µmol photons m^{-2} s^{-1} (50-2000 lux)	White, 60 min	ChemF	(Miller & Nobel, 1972)

Spinach	Isolated chloroplasts in buffer	196±4-212±8;	144±3-144±9	290 µmol photons m^{-2} s^{-1} (900 ft-c)	Red (600-700nm), 3 min	ChemF	(Murakami & Packer, 1970b)
	Isolated chloroplasts in buffer	215±19	175±24	70 klux (6500 ft-c)	Red, 30 s	ChemF	(Sundquist & Burris, 1970)
	Isolated chloroplasts in buffer	250±18	198±7	350 µmol photons m^{-2} s^{-1}	Red (635nm), 5 min	ChemF	(Johnson *et al.*, 2011)
	Isolated thylakoids in buffer	294±7	257-289	150-1700 µmol photons m^{-2} s^{-1}	White, 3 min	SANS	(Nagy *et al.*, 2013)
	Leaf	169±2	149±3	1500 µmol photons m^{-2} s^{-1}	White, 60 min	ChemF	(Yamamoto *et al.*, 2013)
	Leaf infiltrated with D_2O	229	224	1700 µmol photons m^{-2} s^{-1}	White, 5 min	SANS	(Ünnep *et al.*, 2014)
	Leaf , 4 °C	160	157-166	2000 µmol photons m^{-2} s^{-1}	White, 60 min	ChemF	(Yoshioka-Nishimura *et al.*, 2014)
	Leaf	206±5	191±5	200 µmol photons m^{-2} s^{-1}	White, 60 min	ChemF	(Wood *et al.*, 2018)
	Leaf grown in “normal light”	220±24	186±24	350 µmol photons m^{-2} s^{-1}	White, 60 min	ChemF	(Wood *et al.*, 2019)
Sea lettuce	Algae cells *in vivo*	198±10	144±6	290 µmol photons m^{-2} s^{-1} (900 ft-c)	Red (600-700nm), 2 min	ChemF	(Murakami & Packer, 1970a)

Green background - illumination-induced SRD increase,
Orange background - illumination-induced SRD decrease
Navy text - records not included in Fig. 2.

Table S3. Structural thylakoid membrane parameters for Arabidopsis and Ficus obtained from Small Angle Neutron Scattering (SANS) fitting. Scattering length densities, number of thylakoid membrane layers, thylakoid membrane thickness (a double sum of lipid tail and lipid head lengths) and Caillé parameter were fixed throughout the fitting. SRD – stacking repeat distance; SLD – scattering length density.

**Fitted parameters**				
	Arabidopsis Dark	Arabidopsis Light	Ficus Dark	Ficus Light
Scale	0.078	0.057	0.177	0.149
Background	0.039	0.026	0.003	0.003
SRD [Å]	193.1	251.2	221.9	221.4
Partition gap length [Å]	33.0	29.7	29.3	34.3
Power law scaling	20.5	1.4	24.4	26.6
Power law exponent	2.4	3.0	2.3	2.3
**Derived parameters**				
Lumen width [Å]	80.1	141.5	112.6	107.1
Lumen percentage of SRD	41.5	56.3	50.8	48.4
**Fixed parameters**				
Tail Length [Å]	15	15	15	15
Head Length [Å]	5	5	5	5
N layers	5	5	5	5
Caillé parameter	0.01	0.01	0.01	0.01
SLD Tail [10^{-6}/Å^2]	2.2	2.2	2.2	2.2
SLD Head [10^{-6}/Å^2]	2.2	2.2	2.2	2.2
SLD Lumen [10^{-6}/Å^2]	3.2	3.2	3.2	3.2
SLD partition gap [10^{-6}/Å^2]	3.0	3.0	3.0	3.0

References

Anderson JM, Horton P, Kim EH, Chow WS. 2012. Towards elucidation of dynamic structural changes of plant thylakoid architecture. *Philos Trans R Soc Lond B Biol Sci* **367**(1608): 3515-3524.

Bérczi A, Møller IM. 1993. Surface charge density estimation by 9-aminoacridine fluorescence titration: improvements and limitations. *European Biophysics Journal* **22**(3): 177-183.

Chow WS, Barber J. 1980a. 9-Aminoacridine fluorescence changes as a measure of surface charge density of the thylakoid membrane. *Biochimica et Biophysica Acta (BBA) - Bioenergetics* **589**(2): 346-352.

Chow WS, Barber J. 1980b. Salt-dependent changes of 9-aminoacridine fluorescence as a measure of charge densities of membrane surfaces. *Journal of Biochemical and Biophysical Methods* **3**(3): 173-185.

Chuartzman SG, Nevo R, Shimoni E, Charuvi D, Kiss V, Ohad I, Brumfeld V, Reich Z. 2008. Thylakoid membrane remodeling during state transitions in Arabidopsis. *Plant Cell* **20**(4): 1029-1039.

Clausen Casper H, Brooks Matthew D, Li T-D, Grob P, Kemalyan G, Nogales E, Niyogi Krishna K, Fletcher Daniel A. 2014. Dynamic Mechanical Responses of Arabidopsis Thylakoid Membranes during PSII-Specific Illumination. *Biophysical Journal* **106**(9): 1864-1870.

Demmig-Adams B, Muller O, Stewart JJ, Cohu CM, Adams WW. 2015. Chloroplast thylakoid structure in evergreen leaves employing strong thermal energy dissipation. *Journal of Photochemistry and Photobiology B: Biology* **152**: 357-366.

Garty Y, Bussi Y, Levin-Zaidman S, Shimoni E, Kirchhoff H, Charuvi D, Nevo R, Reich Z. 2024. Thylakoid membrane stacking controls electron transport mode during the dark-to-light transition by adjusting the distances between PSI and PSII. *Nat Plants* **10**(3): 512-524.

Guardini Z, Gomez RL, Caferri R, Stuttmann J, Dall'Osto L, Bassi R. 2022. Thylakoid grana stacking revealed by multiplex genome editing of LHCII encoding genes. *bioRxiv*: 2021.2012.2031.474624.

Jakubauskas D, Kowalewska Ł, Sokolova AV, Garvey CJ, Mortensen K, Jensen PE, Kirkensgaard JJK. 2019. Ultrastructural modeling of small angle scattering from photosynthetic membranes. *Scientific Reports* **9**(1): 19405.

Jakubauskas D, Mortensen K, Jensen PE, Kirkensgaard JJK. 2021. Small-Angle X-Ray and Neutron Scattering on Photosynthetic Membranes. *Front Chem* **9**: 631370.

Johnson MP, Brain APR, Ruban AV. 2011. Changes in thylakoid membrane thickness associated with the reorganization of photosystem II light harvesting complexes during photoprotective energy dissipation. *Plant Signaling & Behavior* **6**(9): 1386-1390.

Khatoon M, Inagawa K, Pospísil P, Yamashita A, Yoshioka M, Lundin B, Horie J, Morita N, Jajoo A, Yamamoto Y, et al. 2009. Quality control of photosystem II: Thylakoid unstacking is necessary to avoid further damage to the D1 protein and to facilitate D1 degradation under light stress in spinach thylakoids. *J Biol Chem* **284**(37): 25343-25352.

Kirchhoff H, Hall C, Wood M, Herbstová M, Tsabari O, Nevo R, Charuvi D, Shimoni E, Reich Z. 2011. Dynamic control of protein diffusion within the granal thylakoid lumen. *Proc Natl Acad Sci U S A* **108**(50): 20248-20253.

Li M, Mukhopadhyay R, Svoboda V, Oung HMO, Mullendore DL, Kirchhoff H. 2020. Measuring the dynamic response of the thylakoid architecture in plant leaves by electron microscopy. *Plant Direct* **4**(11): e00280.

Miller MM, Nobel PS. 1972. Light-induced Changes in the Ultrastructure of Pea Chloroplasts in Vivo: Relationship to Development and Photosynthesis. *Plant Physiol* **49**(4): 535-541.

Murakami S, Packer L. 1970a. Light-induced Changes in the Conformation and Configuration of the Thylakoid Membrane of Ulva and Porphyra Chloroplasts in Vivo. *Plant Physiology* **45**(3): 289-299.

Murakami S, Packer L. 1970b. Protonation and chloroplast membrane structure. *J Cell Biol* **47**(2): 332-351.

Mustárdy LA, Machowicz E, Faludi-Dániel Á. 1976. Light-induced structural changes of thylakoids in normal and carotenoid deficient chloroplasts of maize. *Protoplasma* **88**(1): 65-73.

Nagy G, Kovács L, Ünnep R, Zsiros O, Almásy L, Rosta L, Timmins P, Peters J, Posselt D, Garab G. 2013. Kinetics of structural reorganizations in multilamellar photosynthetic membranes monitored by small-angle neutron scattering. *The European Physical Journal E* **36**(7): 69.

Pfeiffer S, Krupinska K. 2005. New Insights in Thylakoid Membrane Organization. *Plant and Cell Physiology* **46**(9): 1443-1451.

Rozak PR, Seiser RM, Wacholtz WF, Wise RR. 2002. Rapid, reversible alterations in spinach thylakoid appression upon changes in light intensity. *Plant, Cell & Environment* **25**(3): 421-429.

Schumann T, Paul S, Melzer M, Dormann P, Jahns P. 2017. Plant Growth under Natural Light Conditions Provides Highly Flexible Short-Term Acclimation Properties toward High Light Stress. *Front Plant Sci* **8**: 681.

Sundquist JE, Burris RH. 1970. Light-dependent structural changes in the lamellar membranes of isolated spinach chloroplasts: Measurement by electron microscopy. *Biochimica et Biophysica Acta (BBA) - Bioenergetics* **223**(1): 115-121.

Tsabari O, Nevo R, Meir S, Carrillo LR, Kramer DM, Reich Z. 2015. Differential effects of ambient or diminished CO2 and O2 levels on thylakoid membrane structure in light-stressed plants. *The Plant Journal* **81**(6): 884-894.

Ünnep R, Nagy G, Markó M, Garab G. 2014. Monitoring thylakoid ultrastructural changes in vivo using small-angle neutron scattering. *Plant Physiology and Biochemistry* **81**: 197-207.

Wood WHJ, Barnett SFH, Flannery S, Hunter CN, Johnson MP. 2019. Dynamic Thylakoid Stacking Is Regulated by LHCII Phosphorylation but Not Its interaction with PSI. *Plant Physiol* **180**(4): 2152-2166.

Wood WHJ, MacGregor-Chatwin C, Barnett SFH, Mayneord GE, Huang X, Hobbs JK, Hunter CN, Johnson MP. 2018. Dynamic thylakoid stacking regulates the balance between linear and cyclic photosynthetic electron transfer. *Nat Plants* **4**(2): 116-127.

Yamamoto Y, Aminaka R, Yoshioka M, Khatoon M, Komayama K, Takenaka D, Yamashita A, Nijo N, Inagawa K, Morita N, et al. 2008. Quality control of photosystem II: impact of light and heat stresses. *Photosynthesis Research* **98**(1): 589-608.

Yamamoto Y, Hori H, Kai S, Ishikawa T, Ohnishi A, Tsumura N, Morita N. 2013. Quality control of Photosystem II: reversible and irreversible protein aggregation decides the fate of Photosystem II under excessive illumination. *Front Plant Sci* **4**: 433.

Yoshioka-Nishimura M, Nanba D, Takaki T, Ohba C, Tsumura N, Morita N, Sakamoto H, Murata K, Yamamoto Y. 2014. Quality Control of Photosystem II: Direct Imaging of the Changes in the Thylakoid Structure and Distribution of FtsH Proteases in Spinach Chloroplasts under Light Stress. *Plant and Cell Physiology* **55**(7): 1255-1265.
